# MANTA Versus Suture-based Closure Devices Following Transcatheter Aortic Valve Replacement: An Updated Meta-analysis

**DOI:** 10.1016/j.jscai.2022.100397

**Published:** 2022-06-30

**Authors:** Ahmad Al-Abdouh, Waiel Abusnina, Mohammed Mhanna, Mahmoud Barbarawi, Ahmad Jabri, Anas Bizanti, Ahmed Abdel-Latif, Andrew M. Goldsweig, Mohamad Alkhouli, Hady Lichaa, Jimmy Kerrigan, Timir K. Paul

**Affiliations:** a Department of Hospital Medicine, University of Kentucky, Lexington, Kentucky; b Department of Cardiology, Creighton University School of Medicine, Omaha, Nebraska; c Department of Medicine, University of Toledo, Toledo, Ohio; d Department of Cardiovascular Medicine, University of Connecticut, Farmington, Connecticut; e Department of Cardiovascular Medicine, Case Western University (Metrohealth), Cleveland, Ohio; f Department of Medicine, Lakeland Regional Hospital, Lakeland, Florida; g Gill Heart and Vascular Institute and Division of Cardiovascular Medicine, University of Kentucky, Lexington, Kentucky; h Division of Cardiovascular Medicine, University of Nebraska Medical Center, Omaha, Nebraska; i Department of Cardiovascular Medicine, Mayo Clinic, Rochester, Minnesota; j Department of Medical Education, University of Tennessee, Nashville, Tennessee

**Keywords:** arteriotomy, MANTA, plug-based, suture-based, vascular closure device

## Abstract

**Background:**

Vascular access closure is essential in large-bore arteriotomy procedures, such as transcatheter aortic valve replacement. ​Suture-based devices are frequently used for vascular access closure. MANTA (Teleflex) is a collagen plug–based device used to achieve hemostasis with evolving efficacy and safety data. This study aimed to evaluate plug-based versus suture-based closure devices following large-bore arteriotomy procedures.

**Methods:**

We conducted a systematic review searching PubMed, Cochrane Library, and ClinicalTrials.gov (inception through November 2021) for studies evaluating plug-based versus suture-based closure devices following large-bore arteriotomy procedures. We performed a meta-analysis comparing the length of stay, device failure, mortality, bleeding, and vascular complications between these 2 types of devices.

**Results:**

Eleven studies (2 randomized controlled trials and 9 observational studies) with a total of 3123 patients were included in this analysis. Compared with suture-based devices, plug-based devices were associated with a significant decrease in the length of stay (standardized mean difference: −0.14; 95% CI, −0.25 to −0.03) and vascular closure device failure (odds ratio, 0.63; 95% CI, 0.44-0.91) following the procedure. There were no significant differences in all-cause mortality, major or minor bleeding, and major or minor vascular complications between plug-based and suture-based closure devices.

**Conclusions:**

Plug-based vascular closure devices were associated with a shorter length of stay and lower risk of device failure following large-bore arteriotomy procedures without differences in mortality, bleeding, or vascular complications than suture-based closure devices.

## Highlights

• MANTA is a collagen plug–based device that achieves hemostasis with evolving efficacy and safety data.
• MANTA was associated with a significant decrease in the length of stay in the hospital and vascular closure device failure.
• No significant differences were found regarding all-cause mortality, bleeding, and vascular complications between MANTA and suture-based closure devices.

## Central Illustration

**Figure:**
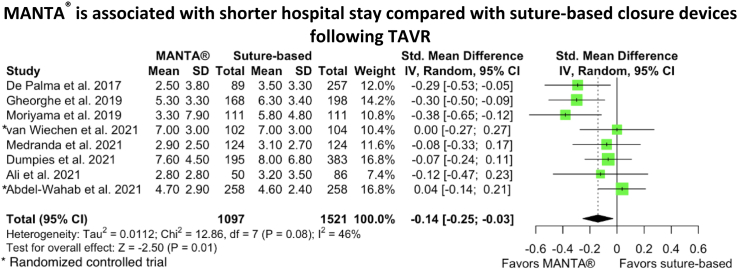

## Introduction

Vascular access closure is essential in large-bore arteriotomy procedures, such as transcatheter aortic valve replacement (TAVR). Large-bore arteriotomy procedures are becoming more prevalent and supplanting surgical procedures in the treatment of various cardiovascular conditions.^1^, ^2^, ^3^ Despite improvements in devices and decreasing sheath diameters, vascular complications remain common, affecting up to 20% of procedures, which contribute to morbidity and mortality.^4^, ^5^, ^6^, ^7^ Vascular complications lead to worse procedural outcomes, and successful closure of large-bore access sites is essential.^8^ Suture-based devices have been the standard method of large-bore closure. ProGlide and Prostar XL are the 2 widely used suture-based vascular closure devices in clinical practice.^9^ A novel collagen plug–based device, MANTA (Teleflex), has become available to address this issue, allowing for postclosure. Plug-based closure devices have demonstrated safety and efficacy in single-arm studies,^10^ but comparative studies versus suture-based closure devices have yielded conflicting results.^11^^,^^12^ There is no contemporary meta-analysis that has aggregated recently published randomized controlled trials (RCTs) and observational studies comparing these 2 vascular closure modalities. We performed a systematic review and meta-analysis using data comparing plug-based and suture-based large-bore vascular closure devices.^13^, ^14^, ^15^, ^16^, ^17^, ^18^, ^19^, ^20^

## Materials and methods

We conducted a meta-analysis based on the Cochrane Collaboration guidelines and according to the Preferred Reporting Items for Systematic Reviews and Meta-analysis methodology.^21^^,^^22^ The protocol of this study was submitted to the International Prospective Register of Systematic Reviews on November 22, 2021; the registration is pending. ​This study was deemed exempt by the institutional review board of the University of Kentucky, as it was a meta-analysis of published studies that included deidentified patient information.

### Data sources and searches

We performed a comprehensive literature search using 3 electronic databases, PubMed, Cochrane library, and ClinicalTrials.gov, from their inception to November 2021. Our search was restricted to English publications. Our search strategy included broad search terms: “plug-based,” “MANTA,” “suture-based,” “Proglide,” “Prostar XL,” “vascular closure device,” “transcatheter aortic valve replacement,” and “TAVR” (Supplemental Table 1). Although plug-based closure may also be used after large-bore vascular access for mechanical circulatory support or endovascular aneurysm repair,^23^ we identified no studies comparing vascular closure devices after these procedures.

### Study selection

**Figure. 1:**
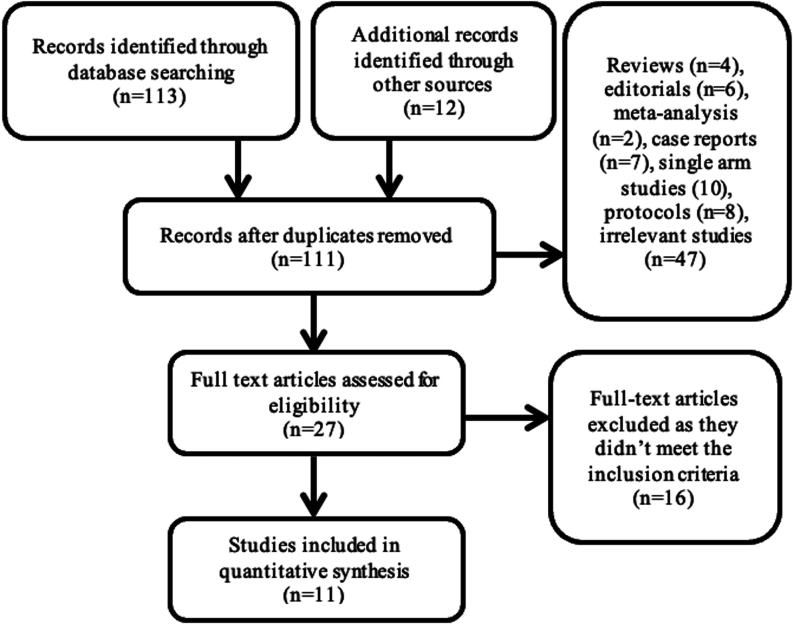
**Details of the search results.** A total of 125 records were identified, 27 full-text articles were assessed for eligibility, and 11 articles were included in the study.

The meta-analysis included the following: (1) all observational studies and RCTs comparing plug-based versus suture-based closure devices following large-bore arteriotomy; and (2) studies with periprocedural outcomes reported, including vascular complications, bleeding, closure device failure, hematoma, pseudoaneurysm, vascular occlusion, and length of stay following the procedure. There were no criteria for the duration of follow-up or sample size of the included studies. Single-arm studies, editorials, and case reports were excluded. The search process and the selection of included studies were performed independently by 2 reviewers (A.A-A. and M.M.). The details of the selection process are outlined in Figure 1.

### Data abstraction, outcomes, and quality assessment

Two reviewers (W.A. and A.A-A.) independently abstracted data from the included studies using prespecified data collection forms, and discrepancies were resolved by a third investigator (M.M.).

We evaluated the quality of the included studies using the Newcastle-Ottawa Scale for observational studies^24^ and the revised Cochrane risk-of-bias tool for the RCTs.^21^ Two authors (M.M. and A.A-A.) assessed each study independently for bias, and all discrepancies were resolved by consensus (Supplemental Table 2). Publication bias was assessed using funnel plots for all outcomes (Supplemental Figure 1). We did not perform the Egger test to evaluate for publication bias, given the number of the studies with reported results in all outcomes was <10.^25^ Leave-one-out analyses were conducted and sorted to identify each trial’s contribution to overall heterogeneity (Supplemental Figure 2).

Outcomes of interest were the postprocedural length of stay, vascular closure device failure, major and minor vascular complications, major and minor bleeding, need for additional intervention, mortality, hematoma, pseudoaneurysm, vascular occlusion, dissection, and need for blood transfusion. Major/minor vascular and bleeding complications were based on the Valve Academic Research Consortium definitions.^26^

### Data synthesis and analysis

The standardized mean difference (SMD) for the length of stay was calculated from the means and SDs in the included studies. When unavailable from the selected studies, means and SDs were calculated as described by Wan et al.^27^ A random-effects model was used to combine odds ratios (ORs) with 95% CIs. We used the Mantel–Haenszel method to estimate τ^2^. The I^2^ statistic was used to measure heterogeneity among the included studies (<25% considered low heterogeneity and >50% considered significant heterogeneity).^28^ Leave-one-out sensitivity analysis was performed to detect any outliners. We performed a sensitivity analysis limited to studies that compared MANTA with ProGlide. Analyses were performed using R Studio version 3.6.3.

## Results

### Summary of studies

We identified 11 studies (2 RCTs and 9 observational studies) for the meta-analysis.^8^^,^^11^, ^12^, ^13^, ^14^, ^15^, ^16^, ^17^, ^18^, ^19^, ^20^ The literature search process is detailed in Figure 1. The details of the included studies are illustrated in Table 1.

**Table 1:** Characteristics of the included studies.

Reference, year	Population	Countries	Type of study	No. of patients	Type of plug-based device	Type of suture-based device	Method to obtain access	Intraprocedural antithrombotic regimen	Reversal of anticoagulation	Follow-up duration
Biancari et al,^11^ 2018	TAVR	Finland	Retrospective observational study	222	MANTA	ProGlide	Fluoroscopy or ultrasound guidance	N/A	N/A	30 ​d
De Palma et al,^18^ 2018	TAVR	Sweden	Prospective observational study	346	MANTA	Prostar XL	Ultrasound with/without fluoroscopic guidance	UFH	Protamine	30 ​d
Hoffmann et al,^8^ 2018	TAVR	Norway	Retrospective observational study	76	MANTA	ProGlide	Fluoroscopy	Intravenous heparin	Protamine	In-hospital
Gheorghe et al,^19^ 2019	TAVR	Netherlands	Retrospective observational study	366	MANTA	Prostar XL	Fluoroscopic or ultrasound guidance	UFH	Protamine	30 ​d
Moriyama et al,^20^ 2019	TAVR	Finland	Retrospective observational study	325	MANTA	ProGlide	Ultrasound guidance	UFH	Protamine	In-hospital
Abdel-Wahab et al,^16^ 2022	TAVR	Germany	RCT	516	MANTA	ProGlide	Angiography or ultrasound guidance	UFH	Protamine	30 ​d
Ali et al,^17^ 2021	TAVR	United Kingdom	Retrospective observational study	136	MANTA	ProGlide	Fluoroscopic or ultrasound guidance	UFH	Protamine	30 ​d
Dumpies et al,^12^ 2021	TAVR	Germany	Retrospective observational study	578	MANTA	ProGlide	Road mapping technique	UFH	Protamine	30 ​d
Medranda et al,^15^ 2021	TAVR	United States	Retrospective observational study	248	MANTA	ProGlide (dual)	Ultrasound guidance	UFH	Protamine	30 ​d
van Wiechen et al,^13^ 2021	TAVR	France and Netherlands	Pilot RCT	210	MANTA	ProGlide	Ultrasound guidance	UFH	Protamine	30 ​d
Sarathy et al,^14^ 2021	TAVR	United Kingdom	Observational cohort study	132	MANTA	ProGlide (dual)	Ultrasound guidance	UFH	Protamine	In-hospital

RCT, randomized controlled trial; N/A, not applicable; TAVR, transcatheter aortic valve replacement; UFH, unfractionated heparin.

A total of 3123 patients were included in our analysis: 1365 in the MANTA group and 1758 in the suture-based devices group. In weighted aggregate, the study population had a mean age of 80.4 ​± ​6.7 ​years in the MANTA group and 80.7 ​± ​7.1 ​years in the suture-based group. There were 46.4% women in the MANTA group and 50.0% women in the suture-based group. The baseline characteristics of the included patients are detailed in Table 2.

**Table 2:** Baseline characteristics of the patients in the included studies.

Reference, year	Treatment arms	No. of patients	Age (y), mean ​± ​SD or median (IQR)	Female	BMI (kg/m^2^), mean ​± ​SD or median (IQR)	Diabetes	Hypertension	Atrial fibrillation	Previous MI	Stroke	PAD	Hemoglobin baseline (g/L), mean ​± ​SD or median (IQR)	Euroscore II, mean ​± ​SD or median (IQR)	STS score, mean ​± ​SD or median (IQR)	Calcification (moderate to severe)
Biancari et al,^11^ 2018	MANTA	107	79.8 ​± ​6.0	66 (61.7%)	27.3 ​± ​4.8	27 (25.2%)	N/A	42 (39.3%)	60 (56.1%)	7 (6.5%)	10 (9.3%)	12.6 ​± ​1.6	4.4 ​± ​3.7	N/A	78 (67.8%)
	ProGlide	115	80.7 ​± ​6.8	63 (54.8%)	28.0 ​± ​5.0	30 (26.1%)	N/A	40 (34.8%)	61 (53.0%)	14 (12.2%)	11 (9.6%)	12.7 ​± ​1.4	4.4 ​± ​3.2	N/A	78 (67.8%)
De Palma et al,^18^ 2018	MANTA	89	81.1 ​± ​6.7	40 (44.9%)	26.8 ​± ​5.1	19 (21.4%)	60 (67.4%)	33 (37.1%)	2 (2.3%)	14 (15.7%)	8 (9.0%)	12.71 ​± ​1.59	4.8 (3.8)	N/A	N/A
	Prostar XL	257	80.7 ​± ​7.2	148 (42.7%)	26.1 ​± ​5.1	61 (23.7%)	196 (76.3%)	98 (38.1%)	8 (3.1%)	18 (7.0%)	51 (19.8%)	12.56 ​± ​1.59	7.2 (7.9)	N/A	N/A
Hoffmann et al,^8^ 2018	MANTA	75	81.2 ​± ​6.5	33 (44.0%)	25.7 ​± ​4.3	15 (20.0%)	54 (72.0%)	N/A	N/A	N/A	N/A	N/A	N/A	2.8 ​± ​1.0	32 (42.7%)
	ProGlide	76	80.8 ​± ​8.3	46 (60.5)	25.6 ​± ​4.7	14 (18.4%)	53 (69.7%)	N/A	N/A	N/A	N/A	N/A	N/A	2.9 ​± ​1.0	48 (63.2%)
Gheorghe et al,^19^ 2019	MANTA	168	80.7 ​± ​6.7	74 (44%)	26.2 ​± ​4.1	36 (22%)	120 (71%)	55 (33%)	26 (16%)	31 (19%)	18 (11%)	N/A	N/A	3.58 ​± ​3.0	18 (10.8%)
	Prostar XL	198	81.7 ​± ​6.1	104 (52%)	26.6 ​± ​4.2	46 (23%)	133 (67%)	83 (42%)	38 (19%)	26 (13%)	25 (13%)	N/A	N/A	4.278 ​± ​2.7	35 (18.6%)
Moriyama et al,^20^ 2019	MANTA	111	79.5 ​± ​7.1	63 (57%)	26.7 ​± ​4.8	30 (27%)	95 (86%)	42 (38%)	N/A	15 (14%)	20 (18%)	12.77 ​± ​1.53	4.4 ​± ​3.3	4.3 ​± ​3.2	N/A
	ProGlide	111	79.8 ​± ​7.2	65 (59%)	27.6 ​± ​5.5	27 (24%)	101 (91%)	48 (43%)	N/A	20 (18%)	18 (16%)	12.63 ​± ​1.6	4.6 ​± ​3.9	4.3 ​± ​2.9	N/A
Abdel-Wahab et al,^16^ 2022	MANTA	258	80.7 ​± ​5.7	115 (44.6%)	28.5 ​± ​5.1	98 (38.0%)	N/A	88 (34.1%)	35 (13.6%)	35 (13.6%)	18 (7.0%)	7.7 ​± ​1.1	4.5 ​± ​4.8	N/A	N/A
	ProGlide	258	80.4 ​± ​6.5	115 (44.6%)	28.6 ​± ​5.3	103 (39.9%)	N/A	71 (27.5%)	29 (11.2%)	31 (12.0%)	21 (8.1%)	7.7 ​± ​1.1	4.6 ​± ​4.3	N/A	N/A
Ali et al,^17^ 2021	MANTA	50	81.5 ​± ​6.8	22 (44%)	28.5 ​± ​6.0	12 (24.0%)	N/A	N/A	3 (6%)	1 (2.0%)	1 (2.0%)	122.4 ​± ​1.6	N/A	N/A	11 (22%)
	ProGlide	86	79.1 ​± ​8.0	37 (43%)	28.2 ​± ​4.6	26 (30.2%)	N/A	N/A	11 (12.8%)	12 (14.0%)	4 (4.7%)	12.18 ​± ​1.6	N/A	N/A	21 (24.7%)
Dumpies et al,^12^ 2021	MANTA	195	80.9 ​± ​6.3	87 (44.6%)	29.2 ​± ​6.1	83 (42.6%)	174 (89.2%)	N/A	20 (10.3%)	27 (13.8%)	21 (10.8%)	7.6 ​± ​1.2	21.2 ​± ​17.8	5.1 ​± ​3.4	43 (22.1%)
	ProGlide	383	80.1 ​± ​6.1	200 (52.2%)	28.8 ​± ​11.8	163 (42.6%)	346 (90.3%)	N/A	59 (15.4%)	41 (10.7%)	36 (9.4%)	7.6 ​± ​1.1	16.4 ​± ​12.1	4.4 ​± ​3.4	76 (19.8%)
Medranda et al,^15^ 2021	MANTA	124	77.5 ​± ​8.7	45 (36.2%)	N/A	51 (41.1%)	109 (87.9%)	32 (25.8%)	16 (12.9%)	6 (4.8%)	13 (10.5%)	12.2 ​± ​2.0	N/A	3.4 ​± ​2.9	41 (36.3%)
	ProGlide	124	76.9 ​± ​9.4	32 (25.8%)	N/A	44 (35.5%)	103 (83.1%)	27 (21.8%)	23 (18.5%)	7 (5.6%)	15 (12.1%)	12.3 ​± ​1.9	N/A	3.5 ​± ​2.6	53 (44.5%)
van Wiechen et al,^13^ 2021	MANTA	102	81 (76-85)	49 (48%)	26 (24-29)	24 (24%)	75 (74%)	N/A	N/A	21 (21%)	6 (6%)	8.0 (7.3-8.9)	2.6 (1.9-3.6)	2.7 (1.8-4.3)	38 (37%)
	ProGlide	104	82 (74-84)	46 (44.2%)	26 (23-29)	23 (22%)	71 (68%)	N/A	N/A	15 (15%)	3 (3%)	7.8 (6.9-8.4)	2.4 (1.6-4.3)	2.8 (1.6-3.9)	43 (43%)
Sarathy et al,^14^ 2021	MANTA	86	80 (77-86)	N/A	27.5 (25.0-30.6)	16 (19%)	70 (82%)	N/A	10 (12%)	N/A	N/A	N/A	N/A	N/A	10 (12%)
	ProGlide	46	86 (78-87)	N/A	26.2 (22.2-31.8)	7 (15%)	32 (69%)	N/A	5 (11%)	N/A	N/A	N/A	N/A	N/A	12 (26%)

BMI, body mass index; MI, myocardial infarction; N/A, not applicable; PAD, peripheral artery disease; STS , Society of Thoracic Surgeons.

### Outcomes

**Figure. 2:**
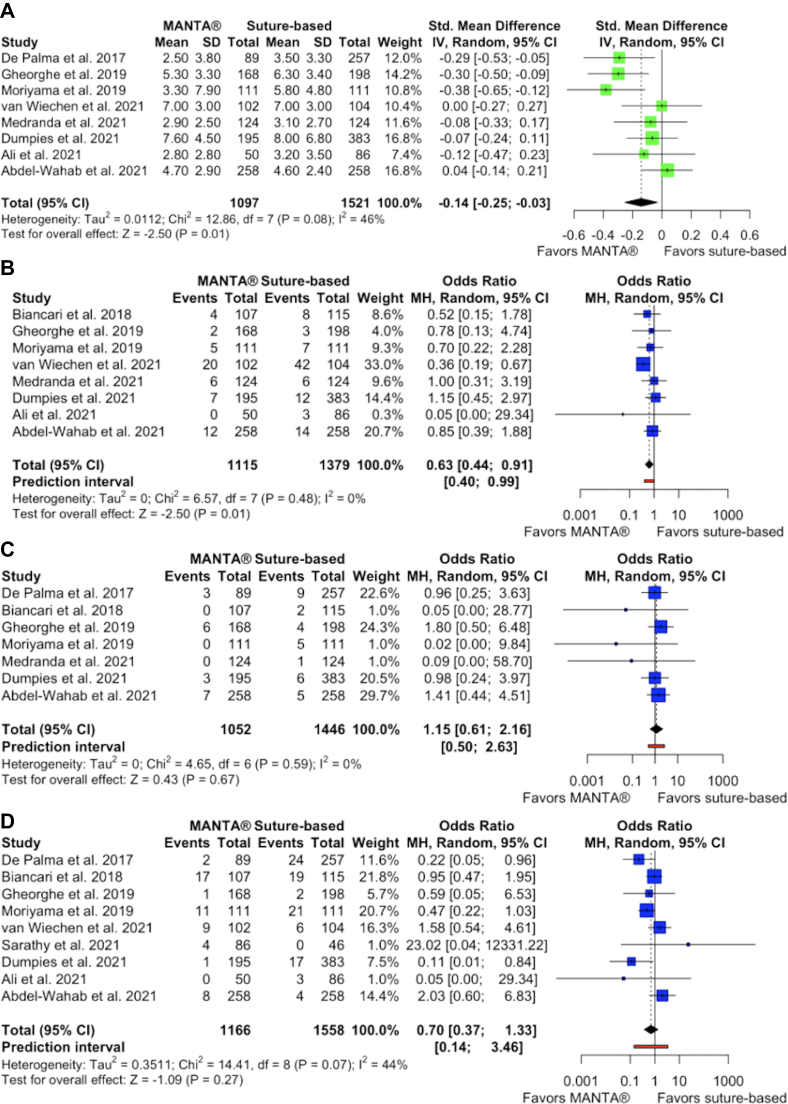

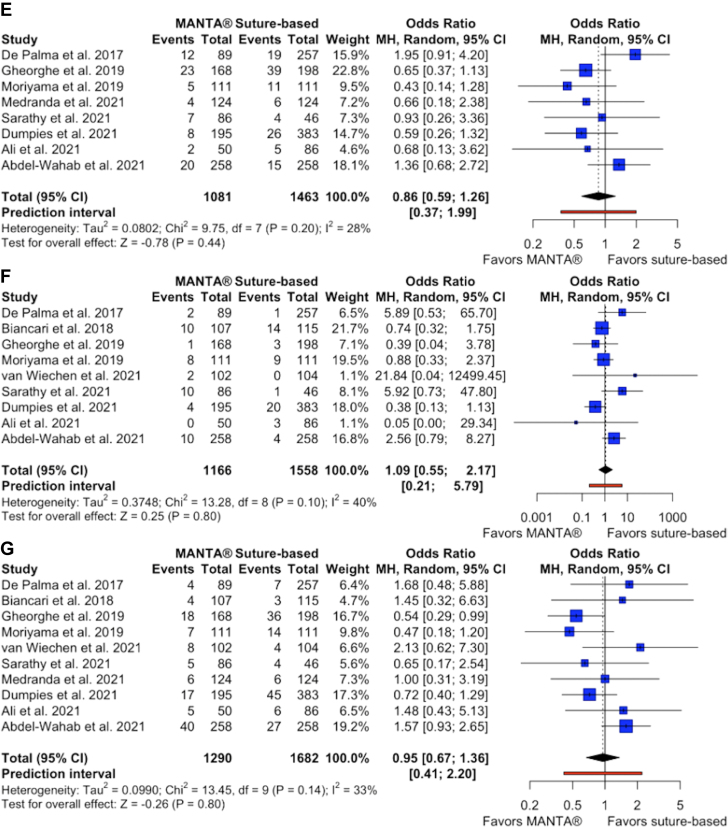
**Forest plots of outcomes.** (**A**) Forest plot of length of stay after the procedure. (**B**) Forest plot of vascular closure device failure. (**C**) Forest plot of all-cause mortality. (**D**) Forest plot of major bleeding. (**E**) Forest plot of minor bleeding. (**F**) Forest plot of major vascular complications. (**G**) Forest plot of minor vascular complications.

Plug-based closure devices were associated with a significantly decreased length of hospital stay following the procedure (SMD, −0.14; 95% CI, −0.25 to −0.03; *P* ​= ​.01; I^2^ ​= ​46%) (Figure 2A, Central Illustration) and less frequent closure device failure (OR, 0.63; 95% CI, 0.44-0.91; *P* ​= ​.01; I^2^ ​= ​0%) (Figure 2B) compared with suture-based devices. There were no significant differences between the groups in terms of all-cause mortality (OR, 1.15; 95% CI, 0.61-2.16; *P* ​= ​.67; I^2^ ​= ​0%), major bleeding (OR, 0.70; 95% CI, 0.37-1.33; *P* ​= ​.27; I^2^ ​= ​44%), minor bleeding (OR, 0.86; 95% CI, 0.59-1.26; *P* ​= ​.44; I^2^ ​= ​28%), major vascular complications (OR, 1.09; 95% CI, 0.55-2.17; *P* ​= ​.80; I^2^ ​= ​40%), or minor vascular complications (OR, 0.95; 95% CI, 0.67-1.36; *P* ​= ​.80; I^2^ ​= ​33%) (Figure 2C-G). Additionally, there were no significant differences in rates of specific vascular complications between the groups, including hematoma (OR, 0.92; 95% CI, 0.42-2.03; *P* ​= ​.83; I^2^ ​= ​75%), flow limiting dissection (OR, 1.03; 95% CI, 0.58-1.83; *P* ​= ​.91; I^2^ ​= ​0%), flow limiting vascular occlusion (OR, 0.98; 95% CI, 0.28-3.40; *P* ​= ​.98; I^2^ ​= ​10%), pseudoaneurysm (OR, 1.78; 95% CI, 0.43-7.41; *P* ​= ​.42; I^2^ ​= ​52%), requirement of blood transfusion (OR, 0.94; 95% CI, 0.47-1.87; *P* ​= ​.86; I^2^ ​= ​58%), or requirement of additional interventions (OR, 1.21; 95% CI, 0.85-1.73; *P* ​= ​.30; I^2^ ​= ​0%) (Supplemental Figure 2A-F).

**Central Illustration:**
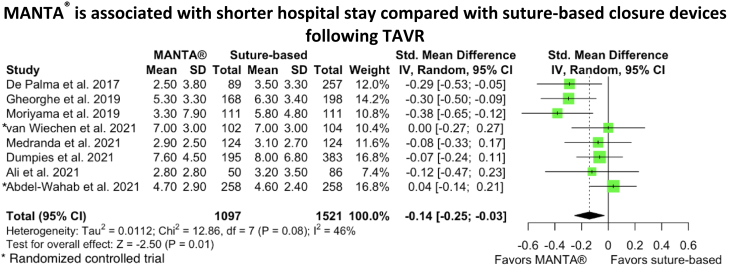
**MANTA is associated with shorter hospital stay compared withsuture-basedclosure devices following transcatheter aortic valve replacement.**

### Sensitivity analyses and heterogeneity evaluation

Omitting the study by van Wiechen et al,^13^ with the highest events and highest weight (33%), made the difference in closure device failure nonsignificant (OR, 0.83; 95% CI, 0.54-1.29; I^2^ ​= ​0%) (Supplemental Figure 3C). Heterogeneity was not substantial among specific vascular complications (Supplemental Figure 3A-M). Three leave-one-out analyses produced I^2^ of >50%:

1. Hematoma: The study by Abdel-Wahab et al^16^ was the study with the highest contribution to heterogeneity, and when excluded, I^2^ decreased from 75% to 46% without substantial changes to the results (OR, 0.68; 95% CI, 0.34-1.33; I^2^ ​= ​46%) (Supplemental Figure 3I).
2. Pseudoaneurysm: The study by Abdel-Wahab et al^16^ was the study with the highest contribution to heterogeneity. When excluded, the I^2^ decreased from 52% to 29% without substantial changes to the results (OR 1.02; 95% CI 0.23-14.56; I^2^ ​= ​29%) (Supplemental Figure 3L).
3. Requiring blood transfusion: The study by Moriyama et al^20^ was the study with the highest contribution to heterogeneity. When excluded, the I^2^ decreased from 58% to 41% without substantial changes to the results (OR, 1.20; 95% CI, 0.64-2.25; I^2^ ​= ​41%) (Supplemental Figure 3M).
  Sensitivity analysis, including only studies that used ProGlide as a suture-based device, showed a significantly lower risk of closure device failure in the MANTA group than in the ProGlide group (OR, 0.64; 95% CI, 0.43-0.94; *P* ​= ​0.02; I^2^ ​= ​8%). There were no significant differences between the groups in other outcomes (Supplemental Figure 4).

### Quality assessment

The quality of the included studies was assessed using the Newcastle-Ottawa Scale for cohort studies^24^ and the revised Cochrane risk-of-bias tool for RCTs,^21^ as shown in Supplemental Table 2. For the Newcastle-Ottawa Scale, each asterisk counts as 1 point. The maximum points are 2 for comparability and 1 for all other categories (Supplemental Table 2). A score of <5 is considered low quality, 5 to 6 is medium quality, and 7 to 9 is high quality. All the included observational studies scored high in quality assessment. The overall risk of bias for the included RCTs was low.

## Discussion

We conducted a systematic review and meta-analysis including 11 studies comparing outcomes between plug-based and suture-based devices for large-bore arteriotomy closure. Compared with suture-based devices, plug-based devices were associated with a significant decrease in the length of stay following the procedure and a decrease in vascular closure device failure. There were no significant differences in mortality, bleeding, or vascular complications.

Since the 1990s, vascular closure devices have been used to achieve hemostasis following large-bore arteriotomy. The primary aims of these devices are to decrease the time to stop bleeding associated with arterial puncture, improve patient comfort, and facilitate early ambulation. Devices are categorized based on their mechanisms as passive and active approximators. Passive approximators deploy a plug without physically closing the puncture site, whereas active approximators close the puncture site using a suture or clip.^29^

There are 2 widely used suture-based closure devices, ProGlide and Prostar XL. The ProGlide device is more frequently used in clinical practice than Prostar XL, which is unavailable in the United States. The Prostar XL device has 4 needles at the ends of 2 polyester sutures, and misfiring of the sutures can lead to device failure and bleeding. The ProGlide device has 1 needle at the end of a polypropylene monofilament suture, which makes it easier to be replaced by a new device in case of misfiring. The suture-based devices need to be in place at the beginning of the procedure before dilating the vessel. On the other hand, the MANTA device can be deployed after the procedure, making it more helpful in case an emergent large-bore vascular access is needed.^30^ The MANTA device is composed of collagen along with an anchoring mechanism that allows the closure of punctures up to 25 French. Its components are resorbed within 6 ​months, except for the suture lock, which can help guide future access away from the prior arteriotomy site.^10^ There are many limitations associated with plug-based closure devices. First, vessel reaccess may be difficult, especially if any vascular complications occur and require surgical cutdown or contralateral balloon occlusion. Second, optimal plug opposition in severely calcified vessels can be challenging as the device toggle may interact with calcium. However, using suture-based devices is also challenging in severely calcified vessels because of the risk of breakage of these devices.^19^^,^^30^ Third, the MANTA device is more expensive than the currently available suture-based devices.^17^

Our meta-analysis showed a shorter length of stay in the hospital when plug-based closure devices were used, and this result persisted in the leave-one-out sensitivity analysis. However, the difference could be clinically not meaningful. We calculated the SMD between the groups, which is a measure of the distance between the groups (equals the mean difference divided by the SD). The SMD between the groups was −0.14 and, in general, it is considered a small effect since it is <0.5.^31^ When we limited our analysis to studies that compared MANTA to ProGlide, there was no significant difference in the length of hospital stay between the groups. Our findings confirm the results of a meta-analysis by Megaly et al^30^ that includes 5 observational studies (3 studies using ProGlide and 2 studies using Prostar XL). The 2 RCTs included in our study (Abdel-Wahab et al^16^ and van Wiechen et al^13^) showed comparable hospital lengths of stay between the groups, which is different from the results of most of the included observational studies that showed shorter length of stay among the MANTA group. This could be due to a selection bias associated with the included observational studies. Additionally, the hospital length of stay seems to be driven by the results of the 3 studies that were conducted earlier (2017-2019), 2 of the studies used Prostar XL, which could be the reason. However, a post hoc analysis of the BRAVO-3 trial (Bivalirudin Versus Heparin Anticoagulation in Transcatheter Aortic Valve Replacement) showed that ProGlide was associated with a lower rate of major and minor vascular complications than Prostar XL.^32^

It was noted that rates of moderate to severe calcification of the access sites were slightly lower for the MANTA group (29.9% vs 32.3%) compared with the suture-based group. As such, the length of stay could be at least partially related to differences in patient complexity in addition to the frequency of device failure.

To our knowledge, the present study is the first to show significantly lower rates of vascular closure device failure with plug-based devices than with suture-based devices. ​It is noteworthy that most included studies reported that most of the operators had more experience with suture-based devices at the time of their studies; thus, the lower device failure in the plug-based group was likely related to the direct effect of the device.^16^^,^^18^^,^^19^ There were 2% to 4% major vascular complications in selected patients and up to 9% in unselected patients with calcified arteries.^33^ The first single-arm study included 50 patients, and only 1 patient developed a major vascular and bleeding complication.^10^ Another single-arm study of 73 patients by Halim et al reported a 13.7% rate of vascular complications (all minor) and a 6.8% incidence of bleeding complications, with only 1.4% classified as major.^34^ The Pivotal Clinical Study to Evaluate the Safety and Effectiveness of the MANTA Percutaneous Vascular Closure Device trial by Wood et al was also a single-arm multicenter study including 341 patients who reported 4.2% major vascular complications and 2.3% major bleeding events.^35^

Several unresolved issues remain regarding the relative safety and efficacy of plug-based and suture-based closure devices. For example, we found that despite a higher rate of device failure in the suture-based arm, bleeding events and vascular complications were not statistically different. It was unclear how most of the studies dealt with device failure, as only a few of the included studies reported what alternative devices were used and/or what procedures were performed in case of device failure, which may affect the hospital length of stay. The ongoing MANTA Versus Suture-based Closure After Transcatheter Aortic Valve Implantation trial (NCT03811119) will help further understand the safety and efficacy of the MANTA device. There is also an ongoing need to evaluate plug-based closure devices in procedures other than TAVR, such as mechanical circulatory support and/or endovascular aneurysm repair.

Our study has several limitations. First, because of the availability of only 2 RCTs, most studies in our meta-analysis were observational and were prone to selection bias and treatment bias.^36^ Second, several outcomes exhibited heterogeneity on leave-one-out sensitivity analyses. Third, means and SDs were calculated for the length-of-stay outcome for a few included studies from the published medians and interquartile ranges, and this calculation assumes a normal distribution of the data, which might be inaccurate.

## Conclusions

Plug-based vascular closure with MANTA was associated with a shorter length of stay and lower chance of device failure following large-bore arteriotomy procedures without significant differences in mortality, bleeding, or vascular complications, compared with suture-based closure devices. Further RCTs are necessary to evaluate the comparative safety and efficacy of plug-based closure devices across the spectrum of patient subgroups, clinical settings, and procedure types.

## References

[1] Greenhalgh R.M., Brown L.C., Kwong G.P.S., Powell J.T., Thompson S.G. (2004). EVAR trial participants. Comparison of endovascular aneurysm repair with open repair in patients with abdominal aortic aneurysm (EVAR trial 1), 30-day operative mortality results: randomised controlled trial. Lancet.

[2] Durko A.P., Osnabrugge R.L., Van Mieghem N.M. (2018). Annual number of candidates for transcatheter aortic valve implantation per country: current estimates and future projections. Eur Heart J.

[3] Prinssen M., Verhoeven E.L.G., Buth J. (2004). A randomized trial comparing conventional and endovascular repair of abdominal aortic aneurysms. N Engl J Med.

[4] Mehilli J., Jochheim D., Abdel-Wahab M. (2016). One-year outcomes with two suture-mediated closure devices to achieve access-site haemostasis following transfemoral transcatheter aortic valve implantation. EuroIntervention.

[5] Bansal A., Kumar A., Jain V. (2021). Impact of hospital procedural volume on use and outcomes of urgent/emergent transcatheter aortic valve replacement. J Am Heart Assoc.

[6] Chieffo A., Van Mieghem N.M., Tchetche D. (2015). Impact of mixed aortic valve stenosis on VARC-2 outcomes and postprocedural aortic regurgitation in patients undergoing transcatheter aortic valve implantation: results from the international multicentric study PRAGMATIC (Pooled Rotterdam-Milan-Toulouse in Collaboration). Catheter Cardiovasc Interv.

[7] Popma J.J., Deeb G.M., Yakubov S.J. (2019). Transcatheter aortic-valve replacement with a self-expanding valve in low-risk patients. N Engl J Med.

[8] Hoffmann P., Al-Ani A., von Lueder T. (2018). Access site complications after transfemoral aortic valve implantation – a comparison of Manta and ProGlide. CVIR Endovasc.

[9] Berti S., Bedogni F., Giordano A. (2020). Efficacy and safety of ProGlide versus Prostar XL vascular closure devices in transcatheter aortic valve replacement: the RISPEVA registry. J Am Heart Assoc.

[10] Van Mieghem N.M., Latib A., van der Heyden J. (2017). Percutaneous plug-based arteriotomy closure device for large-bore access: a multicenter prospective study. JACC Cardiovasc Interv.

[11] Biancari F., Romppanen H., Savontaus M. (2018). MANTA versus ProGlide vascular closure devices in transfemoral transcatheter aortic valve implantation. Int J Cardiol.

[12] Dumpies O., Kitamura M., Majunke N. (2022). Manta versus Perclose ProGlide vascular closure device after transcatheter aortic valve implantation: initial experience from a large European center. Cardiovasc Revasc Med.

[13] van Wiechen M.P., Tchétché D., Ooms J.F. (2021). Suture- or plug-based large-bore arteriotomy closure: a pilot randomized controlled trial. JACC Cardiovasc Interv.

[14] Sarathy K., Patel K.P., Jones D.M. (2021). Large bore vascular access closure device strategies. Struct Hear.

[15] Medranda G.A., Case B.C., Zhang C. (2021). Propensity-matched comparison of large-bore access closure in transcatheter aortic valve replacement using MANTA versus Perclose: a real-world experience. Catheter Cardiovasc Interv.

[16] Abdel-Wahab M., Hartung P., Dumpies O. (2022). Comparison of a pure plug-based versus a primary suture-based vascular closure device strategy for transfemoral transcatheter aortic valve replacement: the CHOICE-CLOSURE randomized clinical trial. Circulation.

[17] Ali N., Dospinescu C., Cunnington M.S., Malkin C.J., Blackman D.J. (2021). A comparison of efficacy, safety and cost between MANTA^TM^ and proglide vascular closure devices following transfemoral transcatheter aortic valve implantation. Heart Res Open J.

[18] De Palma R., Settergren M., Rück A., Linder R., Saleh N. (2018). Impact of percutaneous femoral arteriotomy closure using the MANTA^TM^ device on vascular and bleeding complications after transcatheter aortic valve replacement. Catheter Cardiovasc Interv.

[19] Gheorghe L., Brouwer J., Mathijssen H. (2019). Early outcomes after percutaneous closure of access site in transfemoral transcatheter valve implantation using the novel vascular closure device collagen plug-based MANTA. Am J Cardiol.

[20] Moriyama N., Lindström L., Laine M. (2019). Propensity-matched comparison of vascular closure devices after transcatheter aortic valve replacement using MANTA versus ProGlide. EuroIntervention.

[21] Higgins J.P.T., Altman D.G., Gøtzsche P.C. (2011). The Cochrane Collaboration’s tool for assessing risk of bias in randomised trials. BMJ.

[22] Shamseer L., Moher D., Clarke M. (2015). Preferred reporting items for systematic review and meta-analysis protocols (PRISMA-P) 2015: elaboration and explanation. BMJ.

[23] Moccetti F., Brinkert M., Seelos R. (2019). Insights from a multidisciplinary introduction of the MANTA vascular closure device. JACC Cardiovasc Interv.

[24] Deeks J.J., Dinnes J., D’Amico R. (2003). Evaluating non-randomised intervention studies. Health Technol Assess.

[25] Debray T.P.A., Moons K.G.M., Riley R.D. (2018). Detecting small-study effects and funnel plot asymmetry in meta-analysis of survival data: a comparison of new and existing tests. Res Synth Methods.

[26] Kappetein A.P., Head S.J., Généreux P. (2012). Updated standardized endpoint definitions for transcatheter aortic valve implantation: the Valve Academic Research Consortium-2 consensus document. Eur Heart J.

[27] Wan X., Wang W., Liu J., Tong T. (2014). Estimating the sample mean and standard deviation from the sample size, median, range and/or interquartile range. BMC Med Res Methodol.

[28] Turner R.M., Davey J., Clarke M.J., Thompson S.G., Higgins J.P. (2012). Predicting the extent of heterogeneity in meta-analysis, using empirical data from the Cochrane Database of Systematic Reviews. Int J Epidemiol.

[29] Noori V.J., Eldrup-Jørgensen J. (2018). A systematic review of vascular closure devices for femoral artery puncture sites. J Vasc Surg.

[30] Megaly M., Buda K.G., Brilakis E.S. (2020). Outcomes with MANTA device for large-bore access closure after transcatheter aortic valve replacement: a meta-analysis. Struct Heart.

[31] Faraone S.V. (2008). Interpreting estimates of treatment effects: implications for managed care. P T.

[32] Power D., Schäfer U., Guedeney P. (2019). Impact of percutaneous closure device type on vascular and bleeding complications after TAVR: a post hoc analysis from the BRAVO-3 randomized trial. Catheter Cardiovasc Interv.

[33] Moriyama N., Dahlbacka S., Vähäsilta T. (2019). The efficacy of the ultrasound-navigated MANTA deployment following transfemoral transcatheter aortic valve replacement. JACC Cardiovasc Interv.

[34] Halim J., Missault L., Lycke M., Van der Heyden J. (2020). Assessment of the MANTA closure device in transfemoral transcatheter aortic valve replacement: a single-centre observational study. Neth Heart J.

[35] Wood D.A., Krajcer Z., Sathananthan J. (2019). Pivotal clinical study to evaluate the safety and effectiveness of the MANTA percutaneous vascular closure device. Circ Cardiovasc Interv.

[36] Boyko E.J. (2013). Observational research—opportunities and limitations. J Diabetes Complications.

