# Supplementary material for: MANTA Versus Suture-based Closure Devices Following Transcatheter Aortic Valve Replacement: An Updated Meta-analysis
Source: J Soc Cardiovasc Angiogr Interv. 2022 Jun 30;1(5):100397. doi: 10.1016/j.jscai.2022.100397 (PMC11308113; doi:10.1016/j.jscai.2022.100397)
Supplement: Supplementary Tables 1-3 and Supplementary Figures 1-4 [file mmc1.docx]

**SUPPLEMENTARY APPENDIX**

| **Title** | Page |
| --- | --- |
| **Supplementary Table 1: The search strategy** | 2 |
| **Supplementary Table 2: The risk of bias assessment of the included studies** | 3 |
| **Supplementary Table 3: PRISMA 2009 Checklist** | 4-5 |
| **Supplementary Figure 1: Funnel plots of outcomes** | 6-10 |
| **Supplementary Figure 2: Additional outcomes** | 11-13 |
| **Supplementary Figure 3: Leave-one-out sensitivity analyses** | 14-19 |
| **Supplementary Figure 4: Sensitivity analyses (MANTA^®^ versus Proglide^®^)** | 20-24 |

**Supplementary Table 1: Search Strategy**

| **PubMed** | | |
| --- | --- | --- |
| No. | Search terms | Number of items |
| #1 | "MANTA"[Title/Abstract] OR "plug-based"[Title/Abstract] OR ("pure"[All Fields] AND "plug-based"[Title/Abstract]) OR "plug"[Title/Abstract] | 14,294 |
| #2 | "suture"[Title/Abstract] OR "suture-based"[Title/Abstract] OR "VCD"[Title/Abstract] OR "vascular closure device"[Title/Abstract] OR "vascular closure devices"[MeSH Terms] OR "VCD"[Title/Abstract] OR "Proglide"[Title/Abstract] OR "prostar xl"[Title/Abstract] | 52,386 |
| #3 | "TAVR"[Title/Abstract] OR "transcatheter aortic valve replacement"[Title/Abstract] OR "TAVI"[Title/Abstract] OR "Transcatheter"[Title/Abstract] OR "valve replacement"[Title/Abstract] | 58,561 |
| #5 | #1 AND #2 AND #3 | 45 |
| **Cochrane Library** | | |
| 1 | "MANTA"[All fields] OR "plug-based"[All fields] | 811 |
| #2 | "suture"[All fields] OR "Proglide"[ All fields] OR "prostar xl"[ All fields] | 7,199 |
| #3 | "TAVR"[ All fields] OR "transcatheter aortic valve replacement"[ All fields] | 792 |
| #4 | #1 AND #2 AND #3 | 60 |
| **ClinicalTrials.gov** | | |
| 1 | “vascular closure” OR “transcatheter aortic valve replacement” OR “MANTA” | 8 |

**Supplementary Table 2: The risk of bias assessment of the included studies**

| The Revised Cochrane risk-of-bias tool for randomized trials for randomized controlled trials in the meta-analysis. | | | | | | | | | | | | | | |
| --- | --- | --- | --- | --- | --- | --- | --- | --- | --- | --- | --- | --- | --- | --- |
| Author, year | Bias arising from the randomization process | | Bias due to deviations from intended interventions | | Bias due to missing data | | | Bias in measurement of outcomes | | Bias in selection of the reported result | | low/moderate/serious / critical | | |
| Abdel-Wahab, 2021 | Low | | Low | | Low | | | Moderate | | Low | | Low | | |
| van Wiechen, 2021 | Moderate | | Low | | Low | | | Low | | Low | | Low | | |
| The Newcastle-Ottawa Scale for assessing the quality of nonrandomized studies in the meta-analysis. | | | | | | | | | | | | | | |
|  | Selection | | | | | | | | Outcome | | | | | |
| Study | Representative nest of the exposed cohort | Selection of the non-exposed cohort | | Ascertainment of exposure | | Outcome not present at baseline | Comparability of the cohort | | Assessment of outcome | | Enough follow up duration | | Adequate follow-up | Total score |
| Biancari, 2018 | * | * | | * | | * | ** | | * | | * | | * | 9 |
| De Palma, 2018 | * | * | | * | | * | * | | * | | * | | * | 8 |
| Hoffmann, 2018 | * | * | | * | | * | * | | * | | NA | | * | 7 |
| Moriyama, 2019 | * | * | | * | | * | * | | * | | NA | | * | 7 |
| Gheorghe, 2019 | * | * | | * | | * | * | | * | | * | | * | 8 |
| Sarathy et al. 2021 | * | * | | * | | * | * | | * | | NA | | * | 7 |
| Dumpie, 2021 | * | * | | * | | * | * | | * | | * | | * | 8 |
| Ali, 2021 | * | * | | * | | * | ** | | * | | * | | * | 9 |
| Medranda, 2021 | * | * | | * | | * | ** | | * | | NA | | * | 8 |

Each asterisk represents one star in the Newcastle-Ottawa Scaling System (NOS). The maximum stars are 2 for comparability and 1 are for all other categories. Each star counts towards the total score. Score of 5 to 6 considered as moderate quality and 7 to 9 as high quality.

Abbreviation: NA: not available.

**PRISMA 2009 Checklist**

| **Section/topic** | **#** | **Checklist item** | **Reported on page #** |
| --- | --- | --- | --- |
| **TITLE** | | |  |
| Title | 1 | Identify the report as a systematic review, meta-analysis, or both. | 1 |
| **ABSTRACT** | | |  |
| Structured summary | 2 | Provide a structured summary including, as applicable: background; objectives; data sources; study eligibility criteria, participants, and interventions; study appraisal and synthesis methods; results; limitations; conclusions and implications of key findings; systematic review registration number. | 2 |
| **INTRODUCTION** | | |  |
| Rationale | 3 | Describe the rationale for the review in the context of what is already known. | 3 |
| Objectives | 4 | Provide an explicit statement of questions being addressed with reference to participants, interventions, comparisons, outcomes, and study design (PICOS). | 3 |
| **METHODS** | | |  |
| Protocol and registration | 5 | Indicate if a review protocol exists, if and where it can be accessed (e.g., Web address), and, if available, provide registration information including registration number. | 3 |
| Eligibility criteria | 6 | Specify study characteristics (e.g., PICOS, length of follow-up) and report characteristics (e.g., years considered, language, publication status) used as criteria for eligibility, giving rationale. | 4 |
| Information sources | 7 | Describe all information sources (e.g., databases with dates of coverage, contact with study authors to identify additional studies) in the search and date last searched. | 4 |
| Search | 8 | Present full electronic search strategy for at least one database, including any limits used, such that it could be repeated. | 4 |
| Study selection | 9 | State the process for selecting studies (i.e., screening, eligibility, included in systematic review, and, if applicable, included in the meta-analysis). | 4 |
| Data collection process | 10 | Describe method of data extraction from reports (e.g., piloted forms, independently, in duplicate) and any processes for obtaining and confirming data from investigators. | 4 |
| Data items | 11 | List and define all variables for which data were sought (e.g., PICOS, funding sources) and any assumptions and simplifications made. | 5 |
| Risk of bias in individual studies | 12 | Describe methods used for assessing risk of bias of individual studies (including specification of whether this was done at the study or outcome level), and how this information is to be used in any data synthesis. | 4-5 |
| Summary measures | 13 | State the principal summary measures (e.g., risk ratio, difference in means). | 5 |
| Synthesis of results | 14 | Describe the methods of handling data and combining results of studies, if done, including measures of consistency (e.g., I^2^) for each meta-analysis. | 5 |

| **Section/topic** | **#** | **Checklist item** | **Reported on page #** |
| --- | --- | --- | --- |
| Risk of bias across studies | 15 | Specify any assessment of risk of bias that may affect the cumulative evidence (e.g., publication bias, selective reporting within studies). | 4-5 |
| Additional analyses | 16 | Describe methods of additional analyses (e.g., sensitivity or subgroup analyses, meta-regression), if done, indicating which were pre-specified. | 5 |
| **RESULTS** | | |  |
| Study selection | 17 | Give numbers of studies screened, assessed for eligibility, and included in the review, with reasons for exclusions at each stage, ideally with a flow diagram. | 6 |
| Study characteristics | 18 | For each study, present characteristics for which data were extracted (e.g., study size, PICOS, follow-up period) and provide the citations. | 6 |
| Risk of bias within studies | 19 | Present data on risk of bias of each study and, if available, any outcome level assessment (see item 12). | 8 |
| Results of individual studies | 20 | For all outcomes considered (benefits or harms), present, for each study: (a) simple summary data for each intervention group (b) effect estimates and confidence intervals, ideally with a forest plot. | 6-8 |
| Synthesis of results | 21 | Present results of each meta-analysis done, including confidence intervals and measures of consistency. | 6-7 |
| Risk of bias across studies | 22 | Present results of any assessment of risk of bias across studies (see Item 15). | 8 |
| Additional analysis | 23 | Give results of additional analyses, if done (e.g., sensitivity or subgroup analyses, meta-regression [see Item 16]). | 7-8 |
| **DISCUSSION** | | |  |
| Summary of evidence | 24 | Summarize the main findings including the strength of evidence for each main outcome; consider their relevance to key groups (e.g., healthcare providers, users, and policy makers). | 8 |
| Limitations | 25 | Discuss limitations at study and outcome level (e.g., risk of bias), and at review-level (e.g., incomplete retrieval of identified research, reporting bias). | 11-12 |
| Conclusions | 26 | Provide a general interpretation of the results in the context of other evidence, and implications for future research. | 12 |
| **FUNDING** | | |  |
| Funding | 27 | Describe sources of funding for the systematic review and other support (e.g., supply of data); role of funders for the systematic review. | 5 |

*From:*  Moher D, Liberati A, Tetzlaff J, Altman DG, The PRISMA Group (2009). Preferred Reporting Items for Systematic Reviews and Meta-Analyses: The PRISMA Statement. PLoS Med 6(7): e1000097. doi:10.1371/journal.pmed1000097

For more information, visit: **www.prisma-statement.org**.

**Supplementary figure 1-A: Funnel plot of length of stay**

**
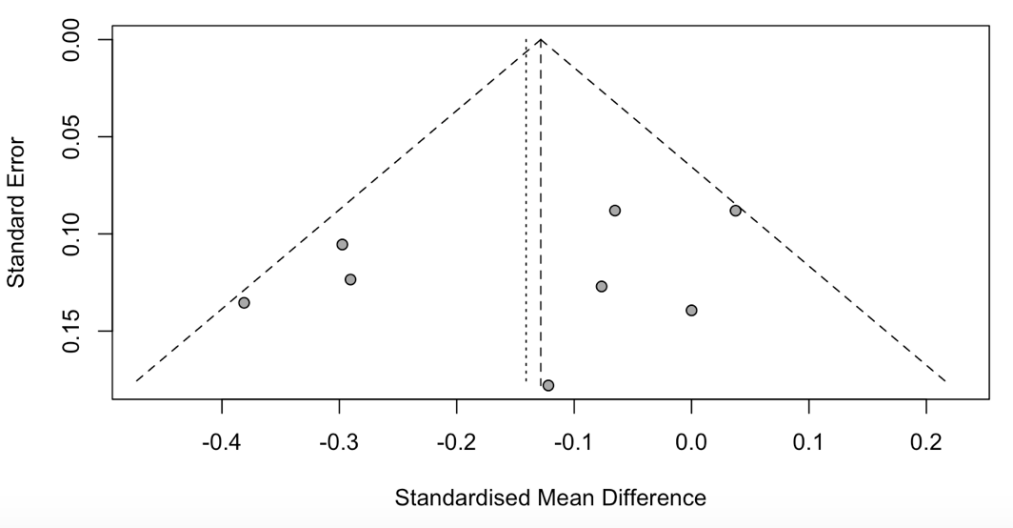
**

**Supplementary figure 1-B: Funnel plot of vascular closure device failure**

**
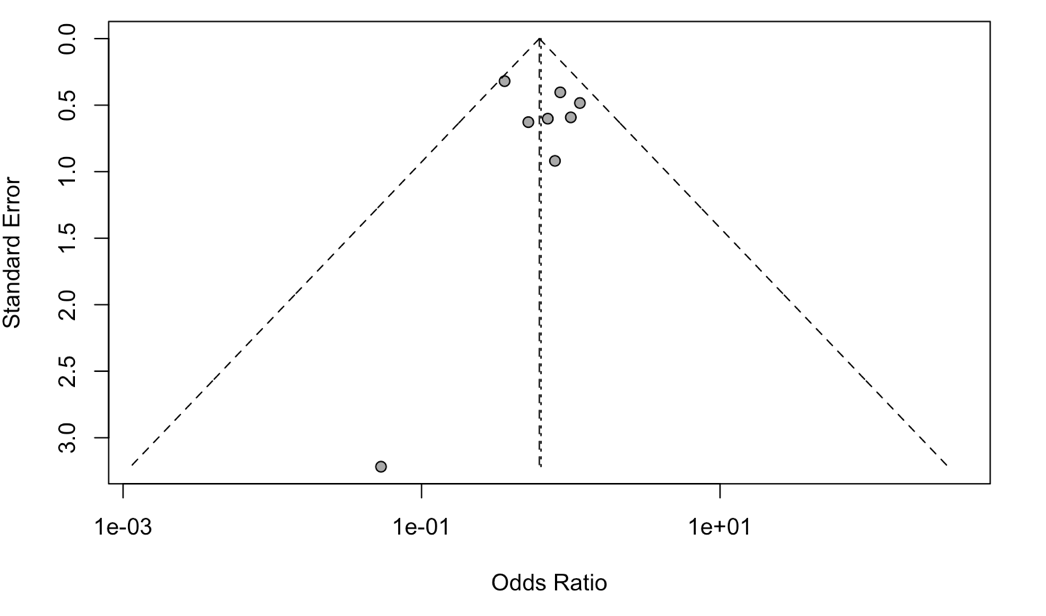
**

**Supplementary figure 1-C: Funnel plot of all-cause mortality**

**
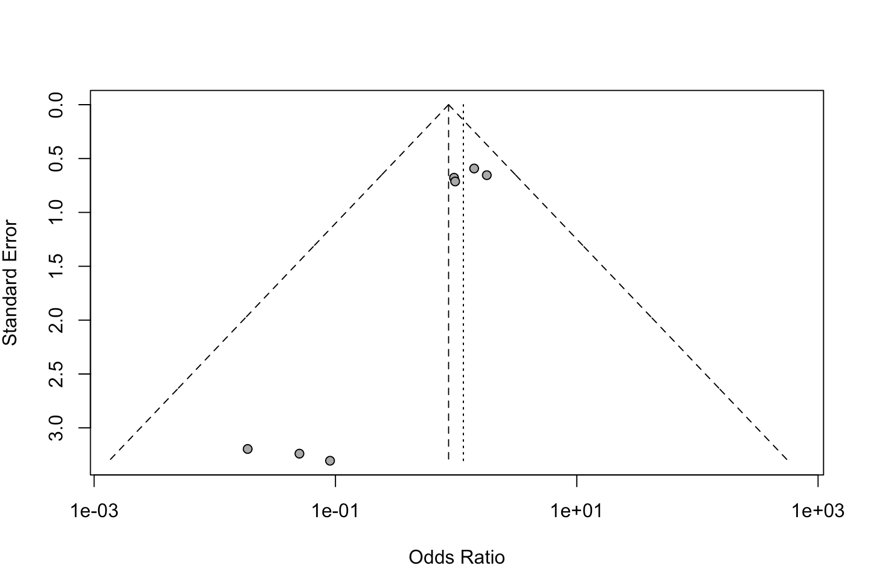
**

**Supplementary figure 1-D: Funnel plot of major bleeding**

**
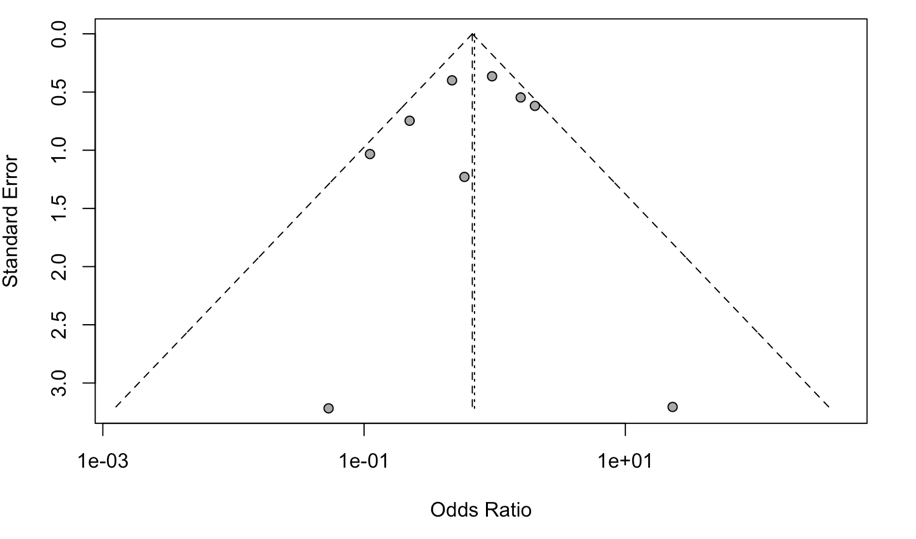
**

**Supplementary figure 1-E: Funnel plot of minor bleeding**

**
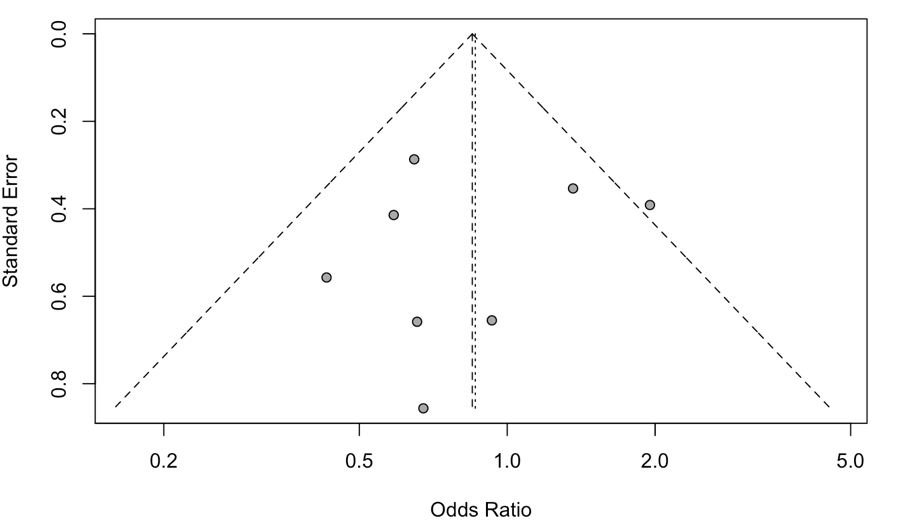
**

**Supplementary figure 1-F: Funnel plot of major vascular complications**

**
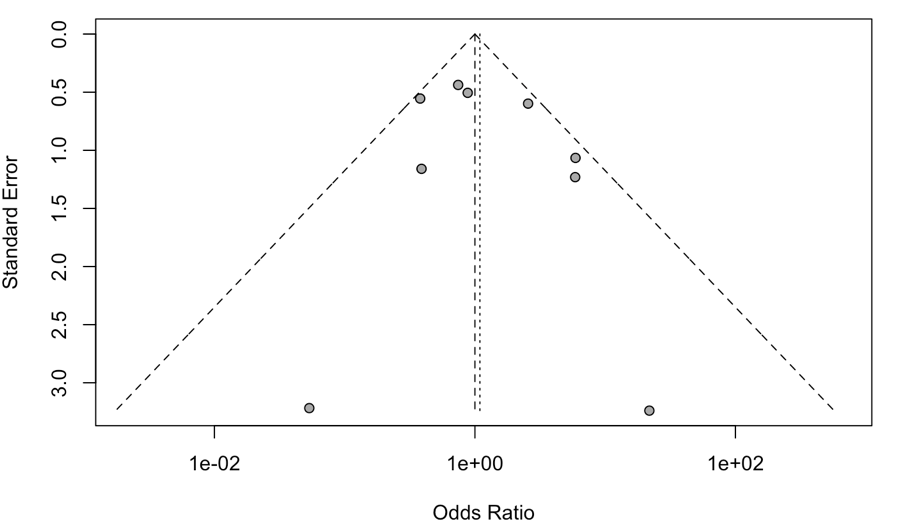
**

**Supplementary figure 1-G: Funnel plot of minor vascular complications**

**
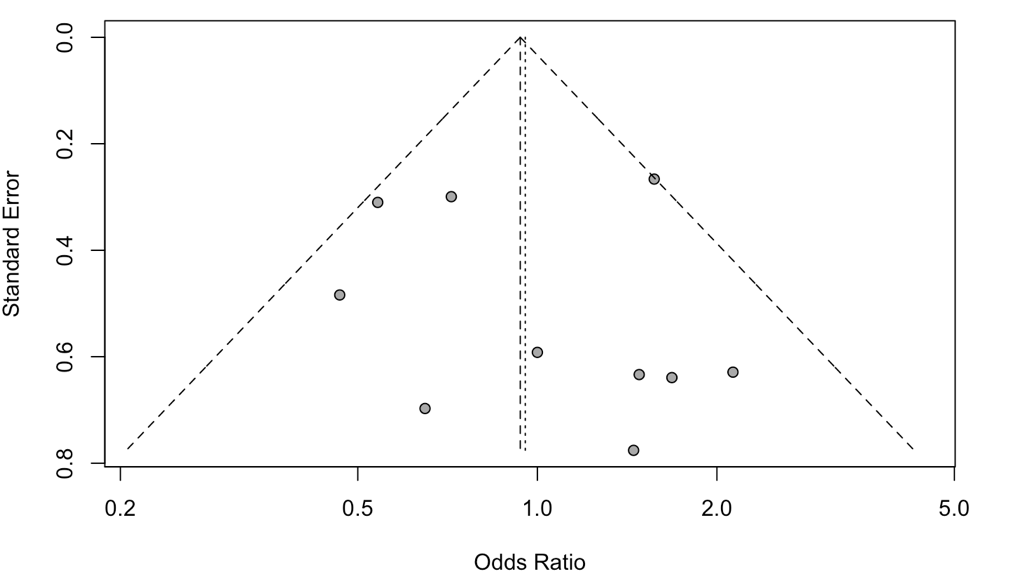
**

**Supplementary figure 1-H: Funnel plot of additional vascular interventions**


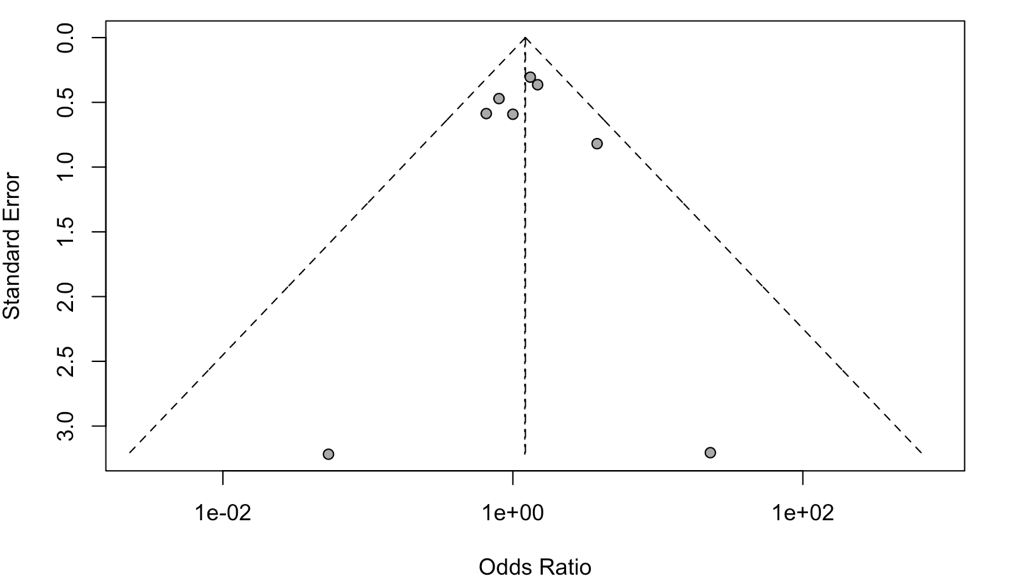


**Supplementary figure 1-I: Funnel plot of hematoma**

**
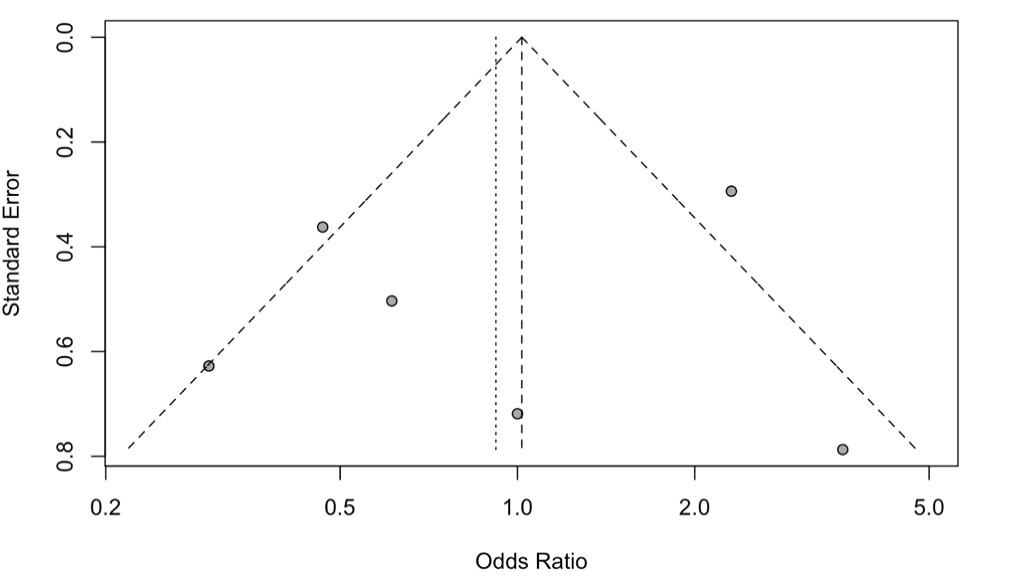
**

**Supplementary figure 1-J: Funnel plot of flow limiting dissection**


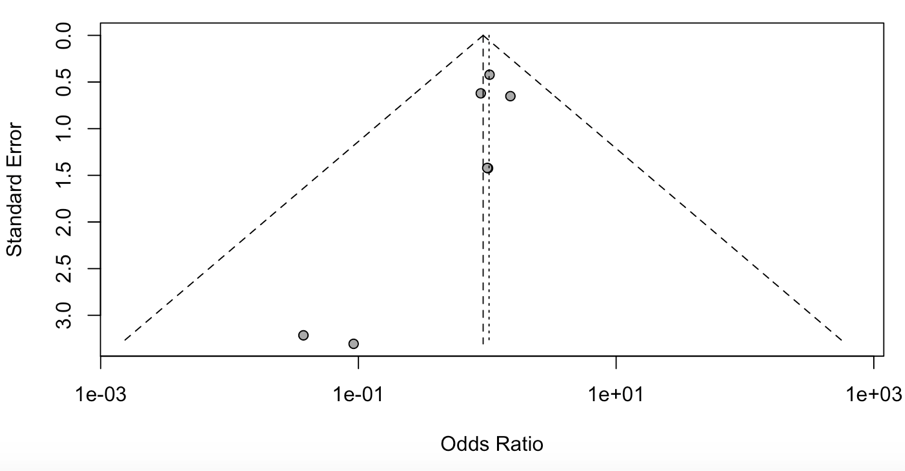


**Supplementary figure 1-K: Funnel plot of flow limiting vascular occlusion**


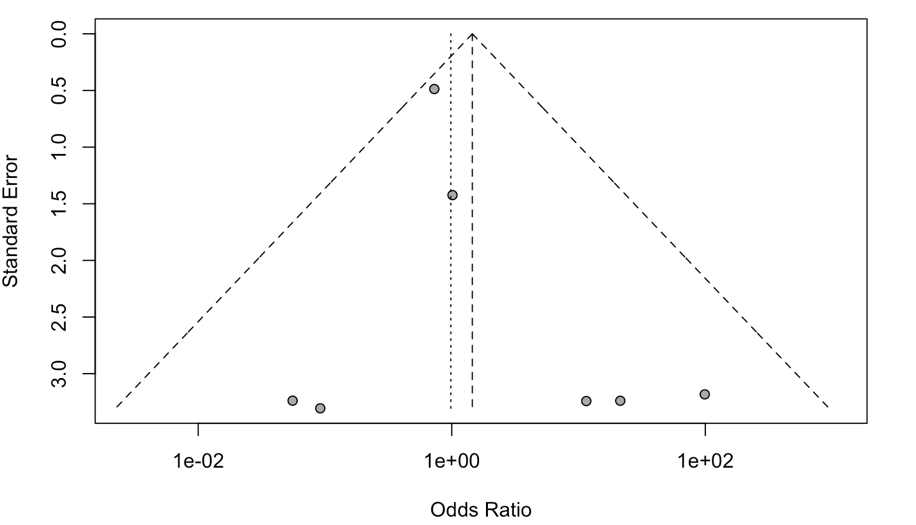


**Supplementary figure 1-L: Funnel plot of pseudoaneurysm**

**
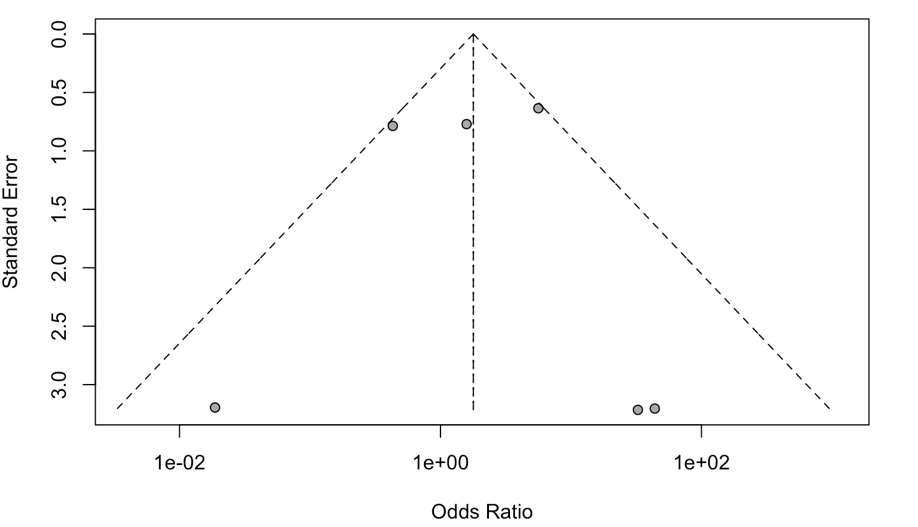
**

**Supplementary figure 1-M: Funnel plot of requiring bleed transfusion**

**
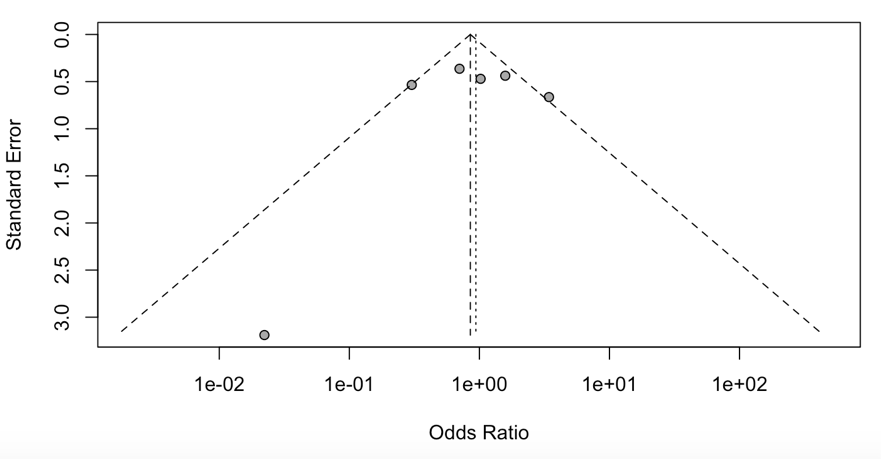
**

**Supplementary Figure 2-A: forest plot of hematoma**


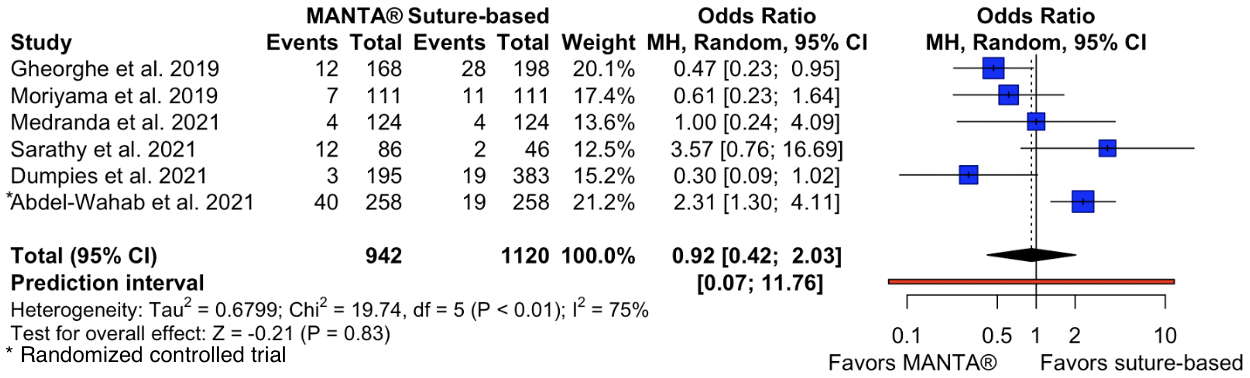


**Supplementary Figure 2-B: forest plot of flow limiting dissection**


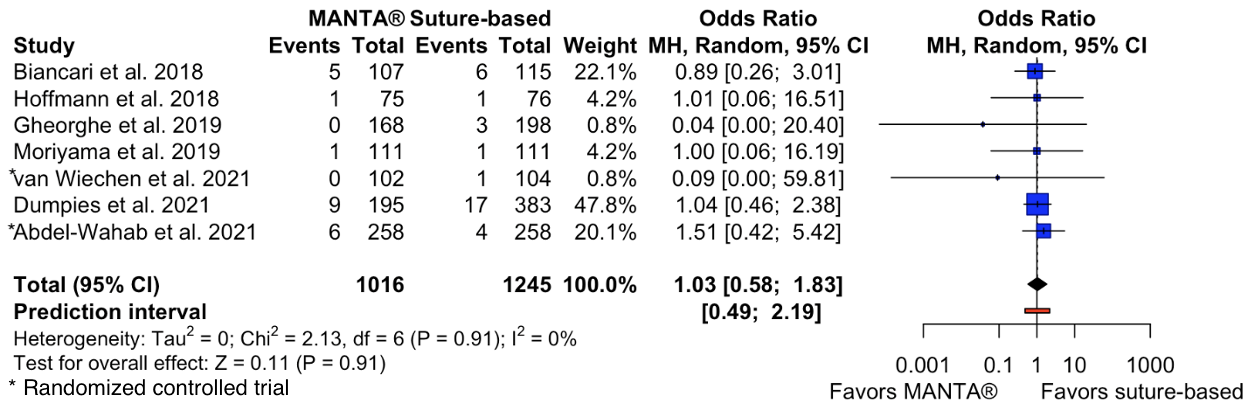


**Supplementary Figure 2-C: forest plot of flow limiting vascular occlusion**


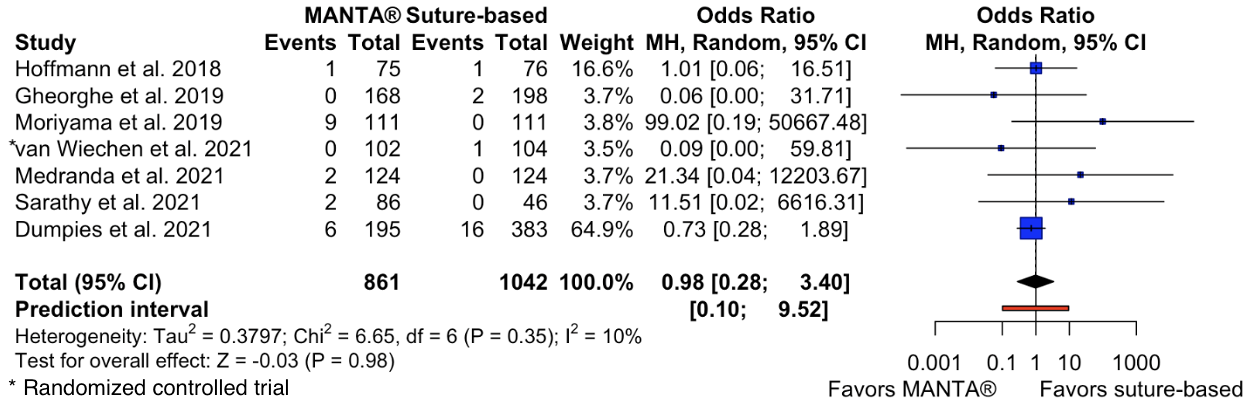


**Supplementary Figure 2-D: forest plot of pseudoaneurysm**

**
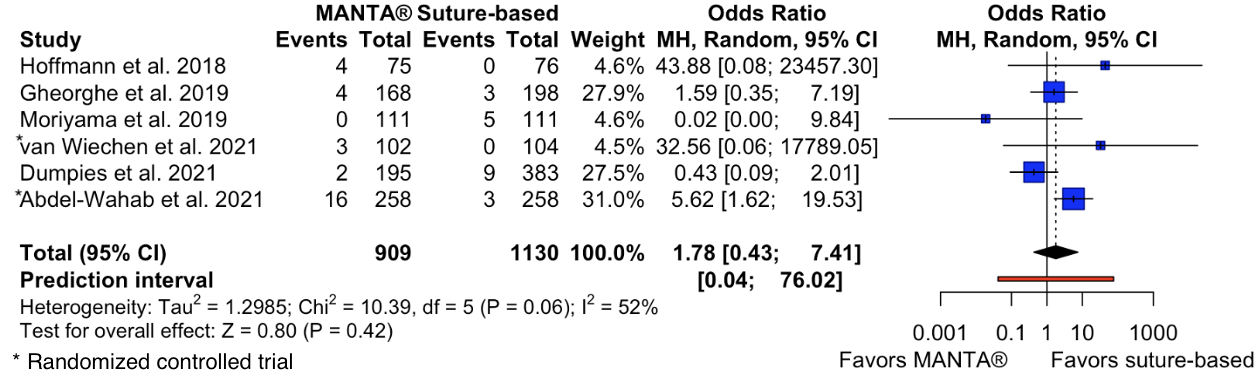
**

**Supplementary Figure 2-E: forest plot of requiring bleed transfusion**


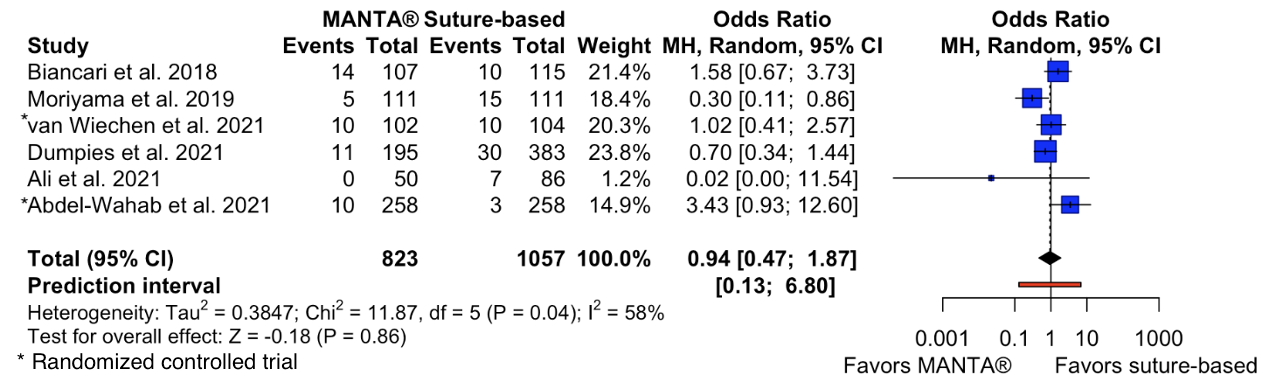


**Supplementary Figure 2-F: forest plot of additional vascular interventions**


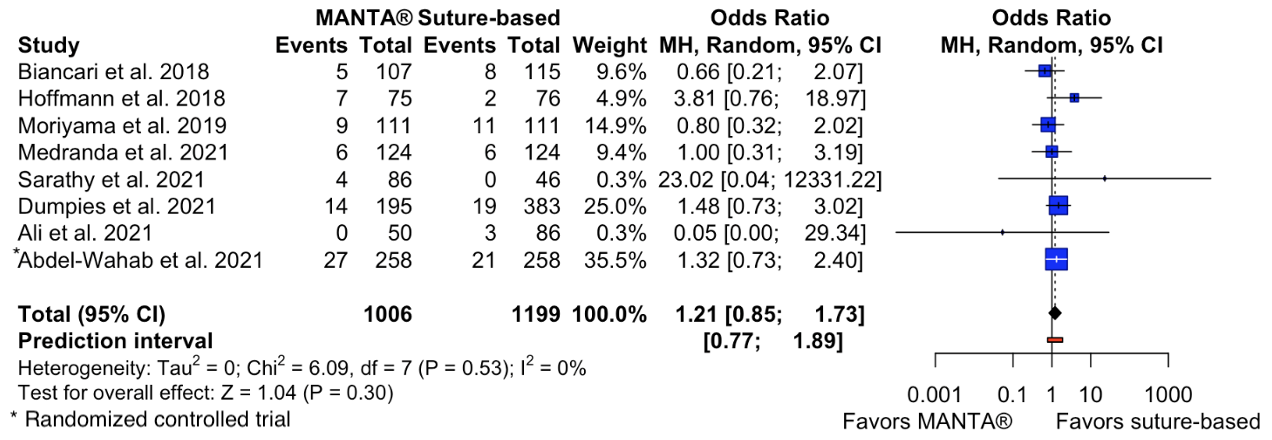


**Supplementary Figure 3-A: Leave-one out sensitivity analyses of length of stay**


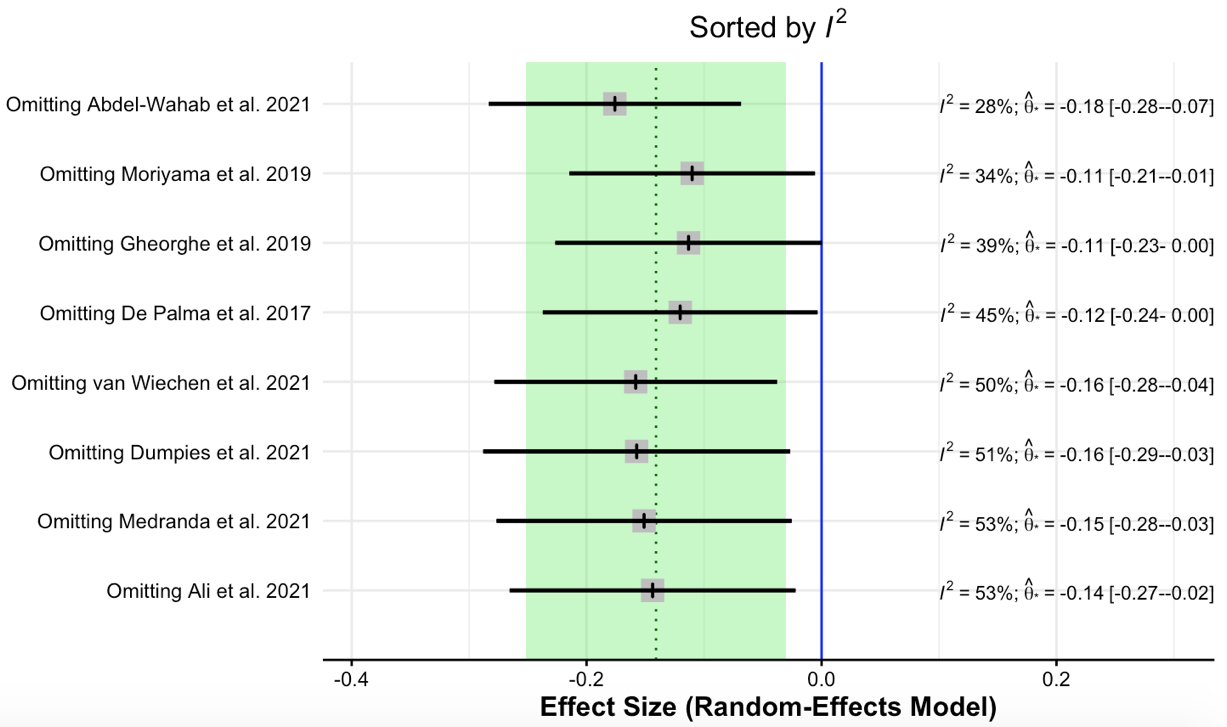


**Supplementary Figure 3-B: Leave-one out sensitivity analyses of vascular closure device failure**


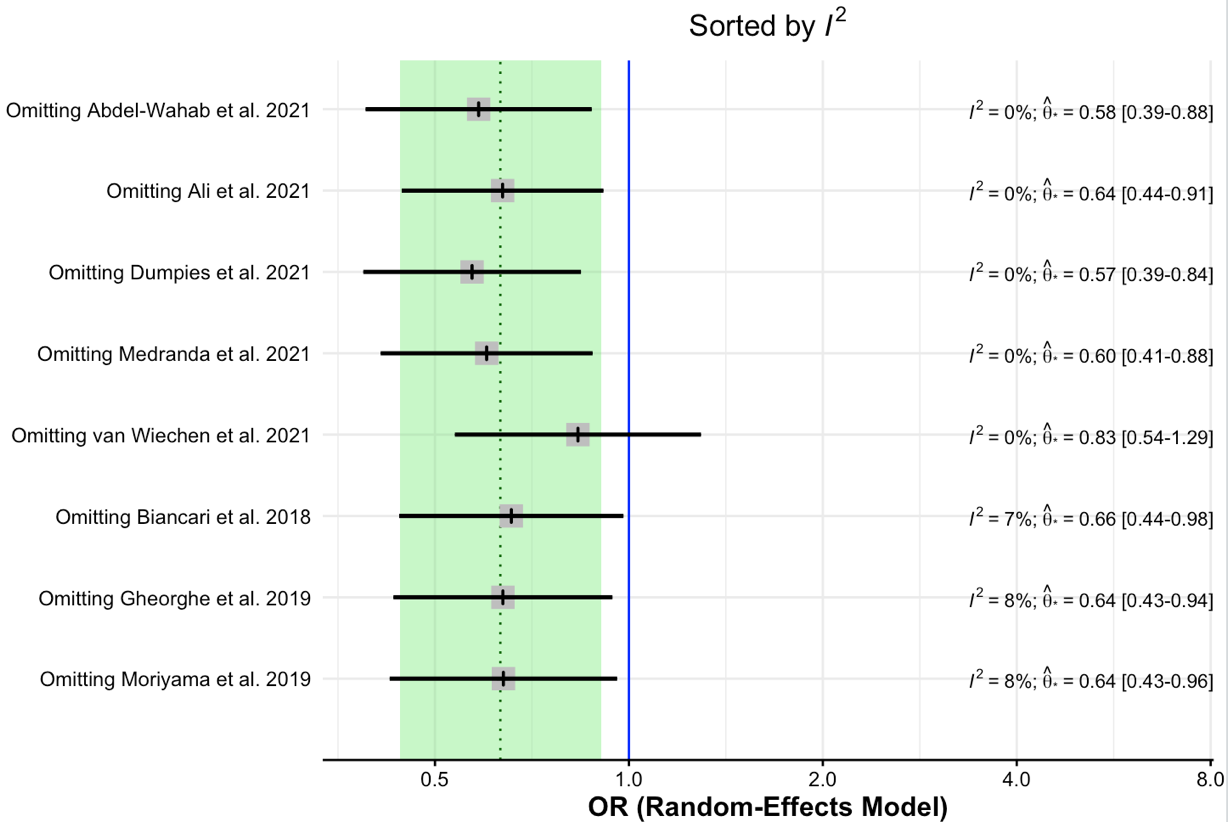


**Supplementary Figure 3-C: Leave-one out sensitivity analyses of mortality**


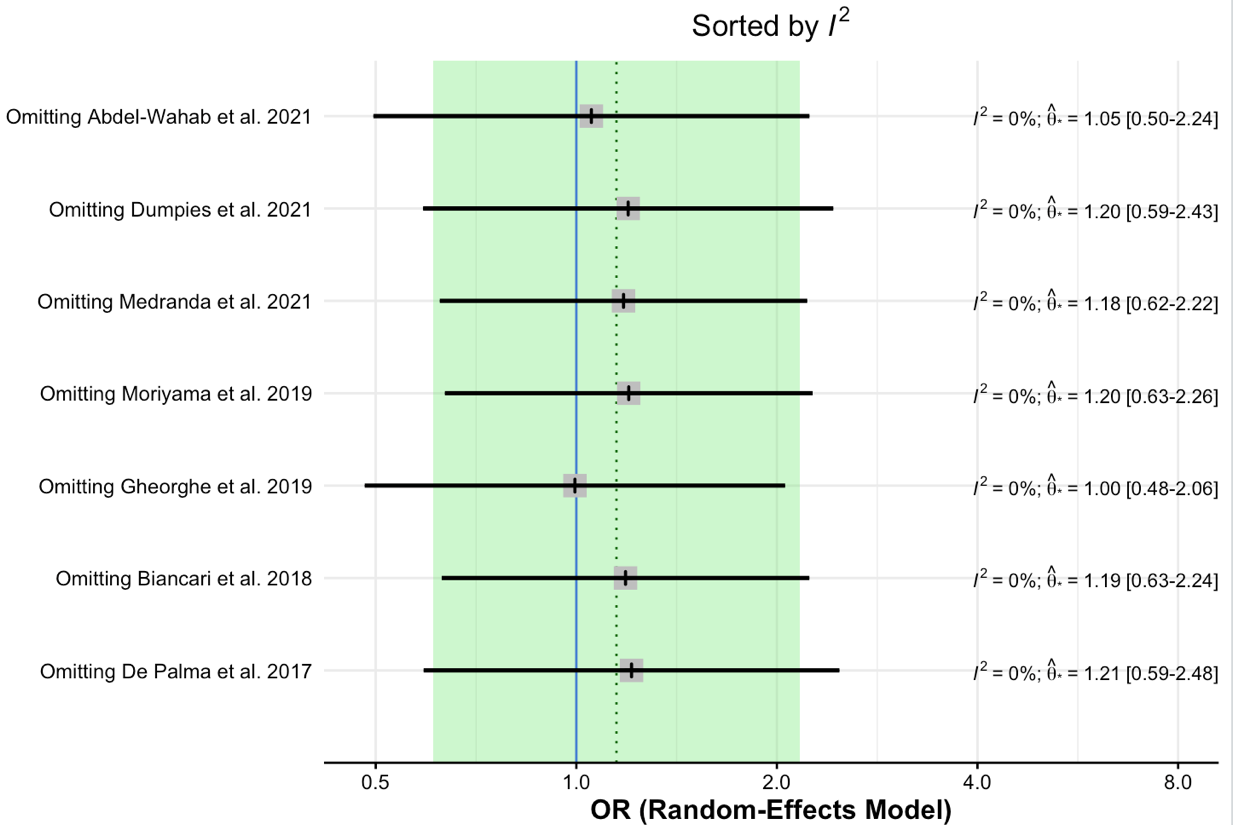


**Supplementary figure 3-D: Leave-one out sensitivity analyses of major bleeding**

**
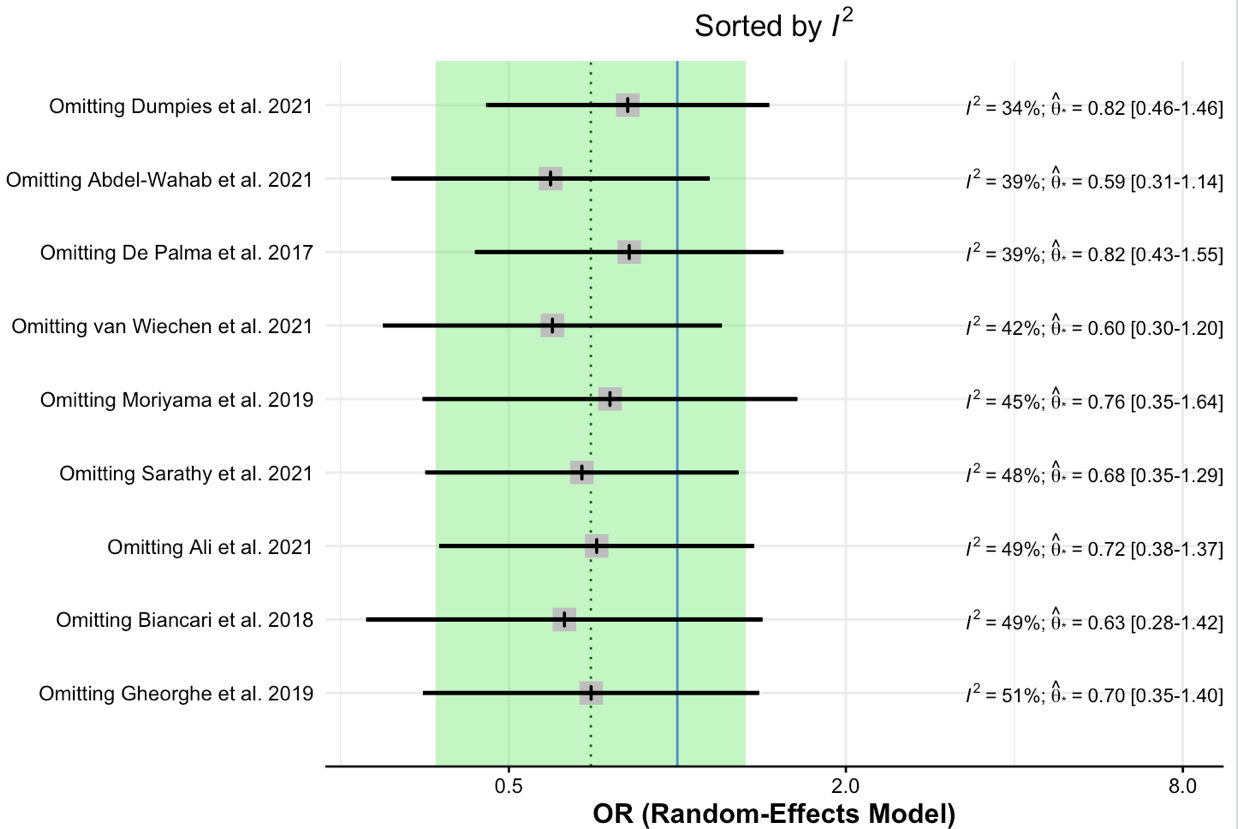
**

**Supplementary figure 3-E: Leave-one out sensitivity analyses of minor bleeding**


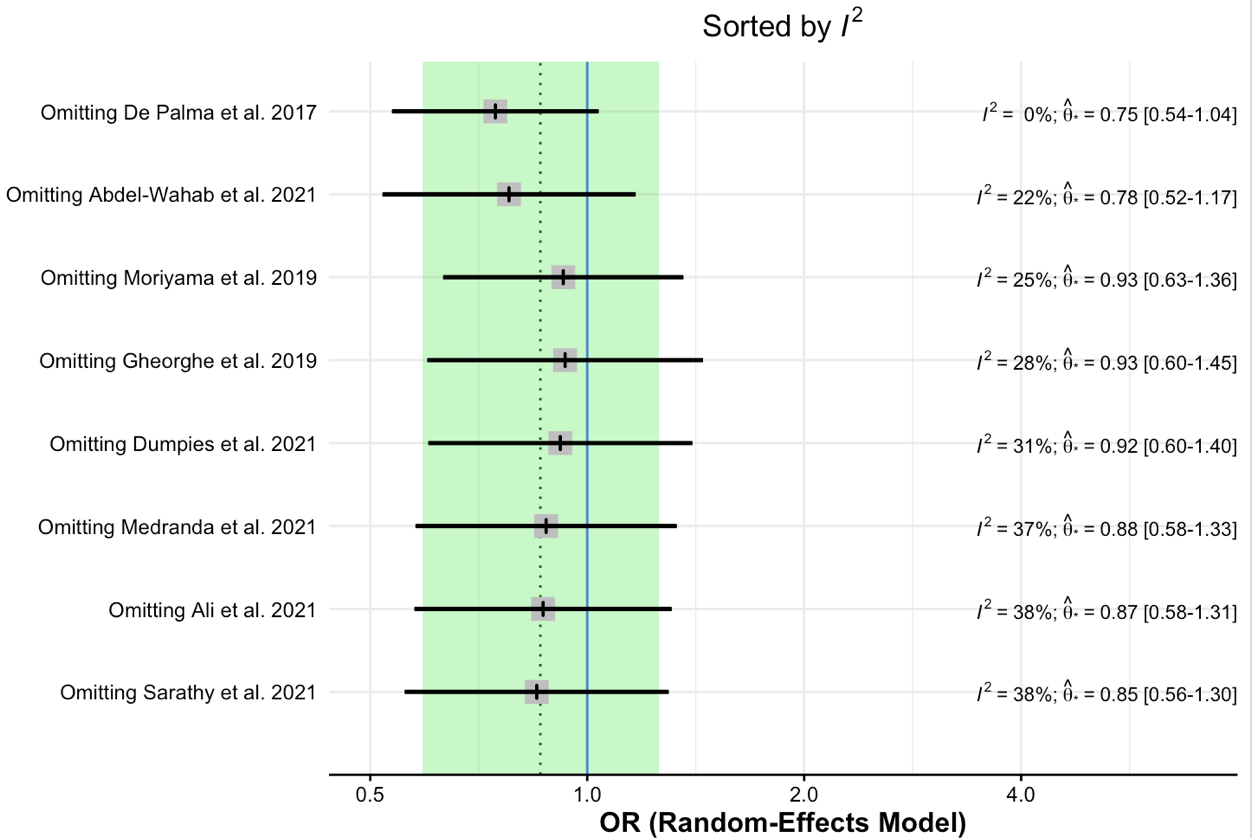


**Supplementary Figure 3-F: Leave-one out sensitivity analyses of major vascular complications**


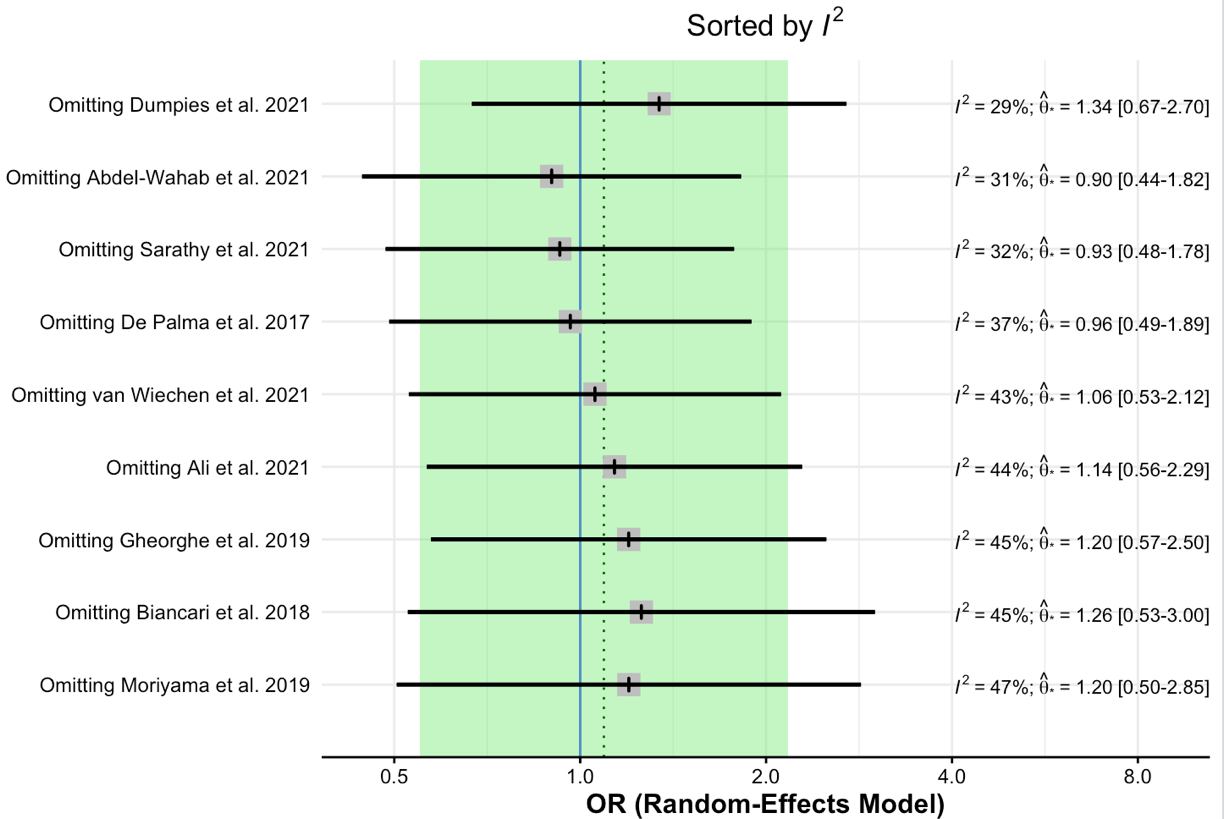


**Supplementary Figure 3-G: Leave-one out sensitivity analyses of minor vascular complications**

**
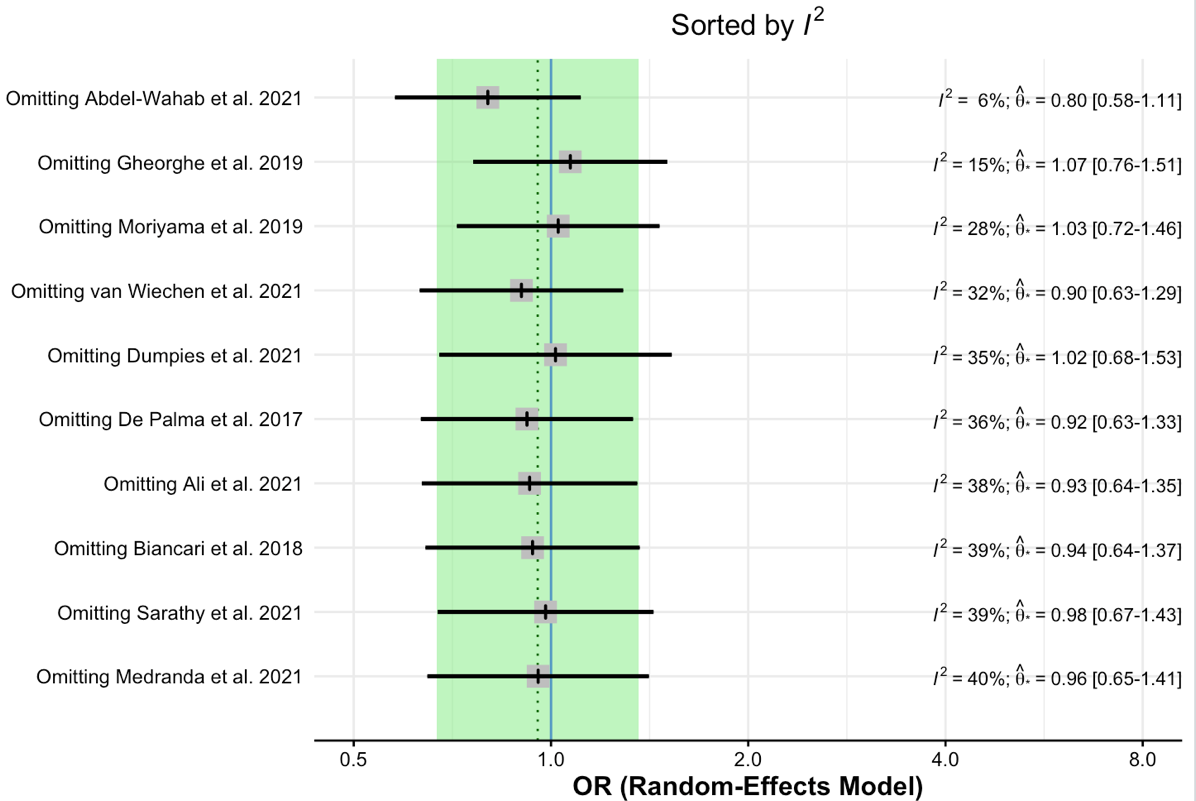
**

**Supplementary Figure 3-H: Leave-one out sensitivity analyses of additional intervention**

**
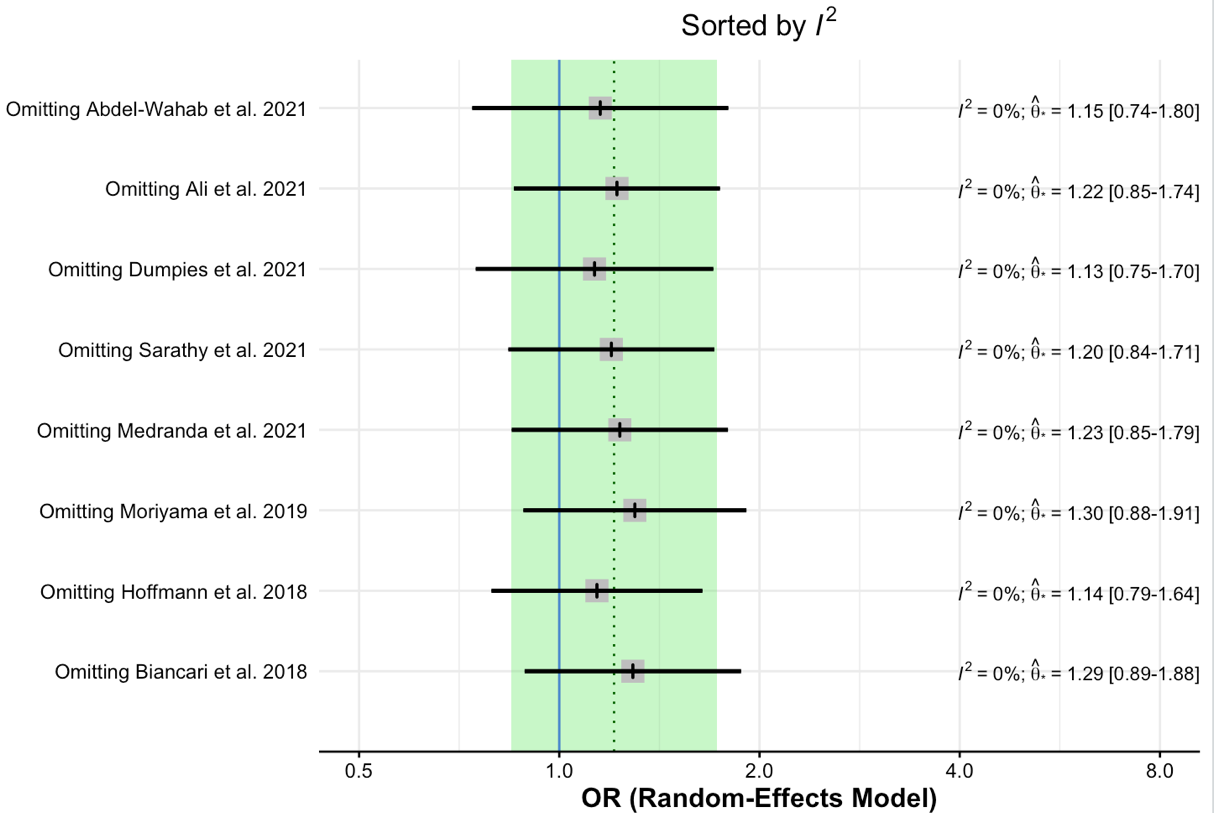
**

**Supplementary Figure 3-I: Leave-one out sensitivity analyses of hematoma**

**
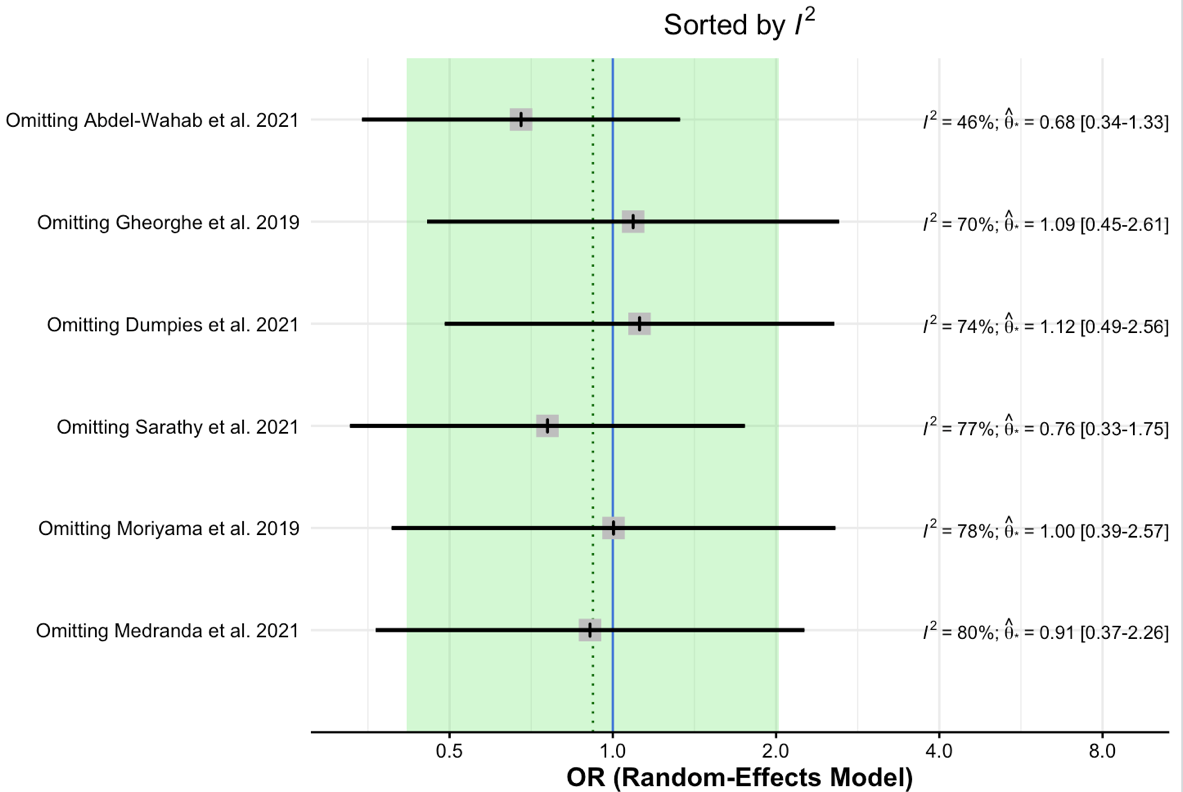
**

**Supplementary Figure 3-J: Leave-one out sensitivity analyses of flow limiting dissection**

**
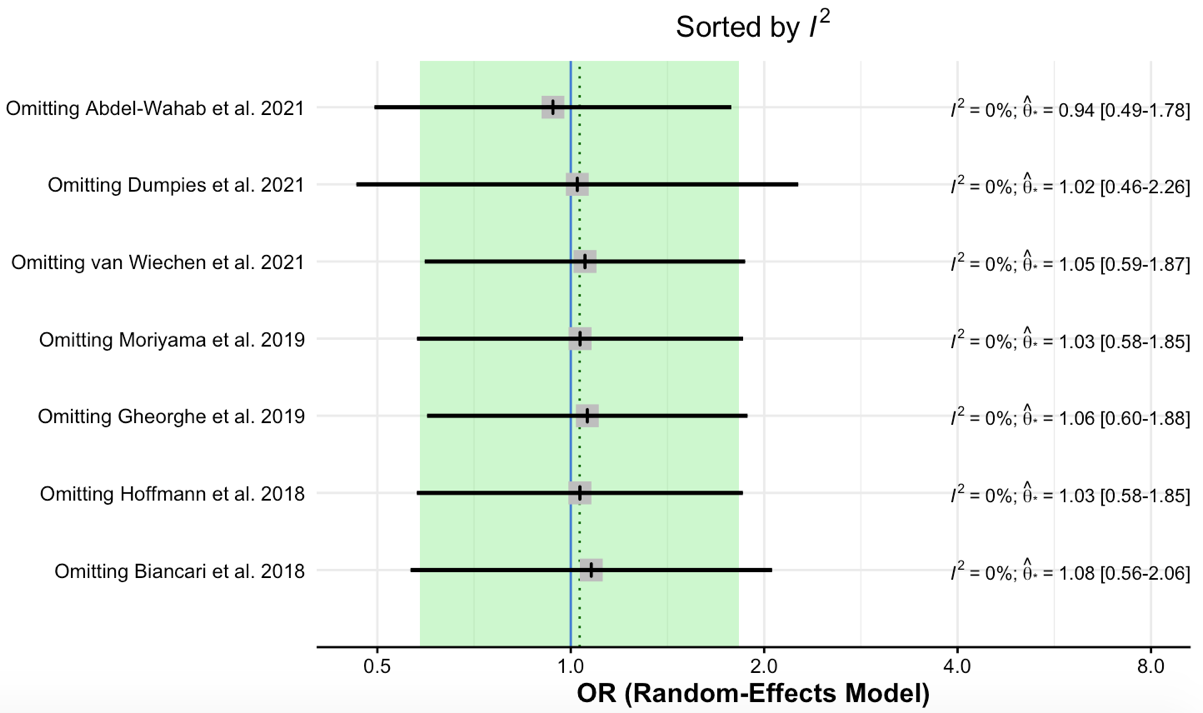
**

**Supplementary Figure 3-K: Leave-one out sensitivity analyses of flow limiting occlusion**

**
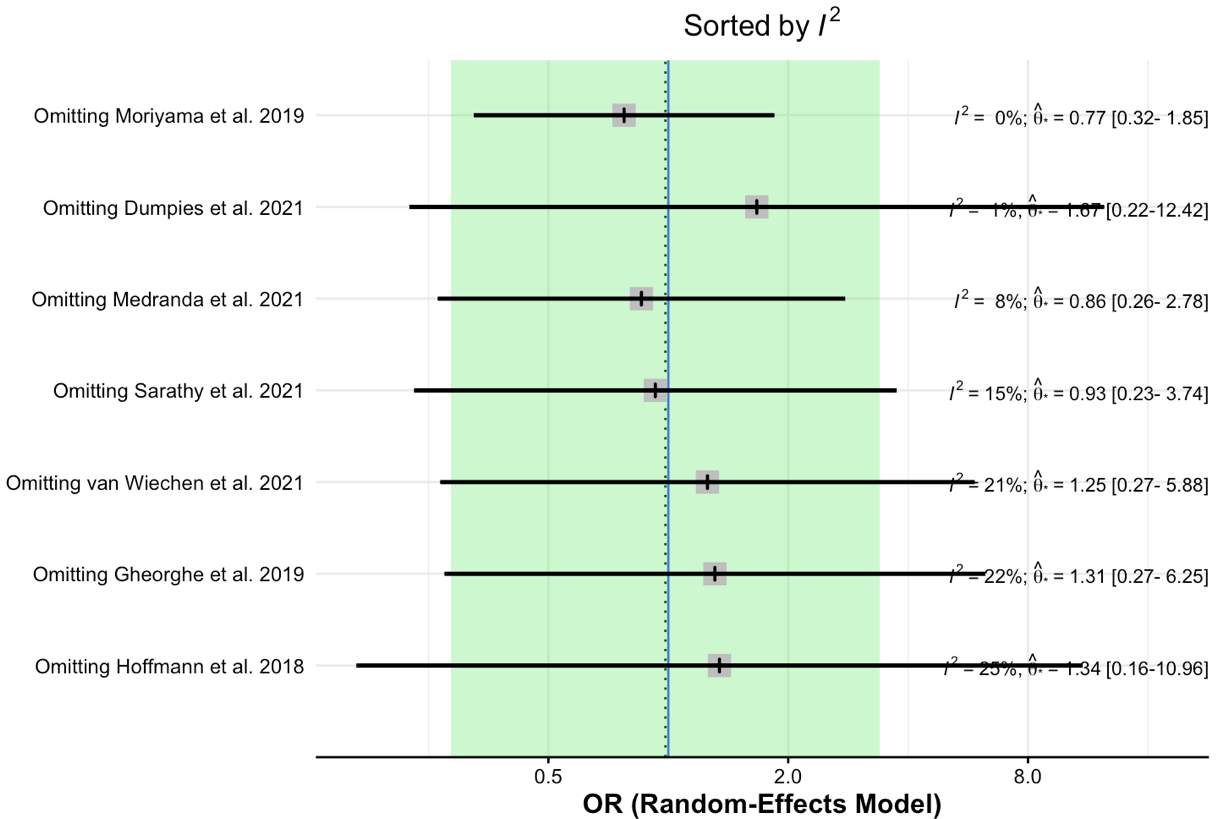
**

**Supplementary Figure 2-L: Leave-one out sensitivity analyses of pseudoaneurysm**

**
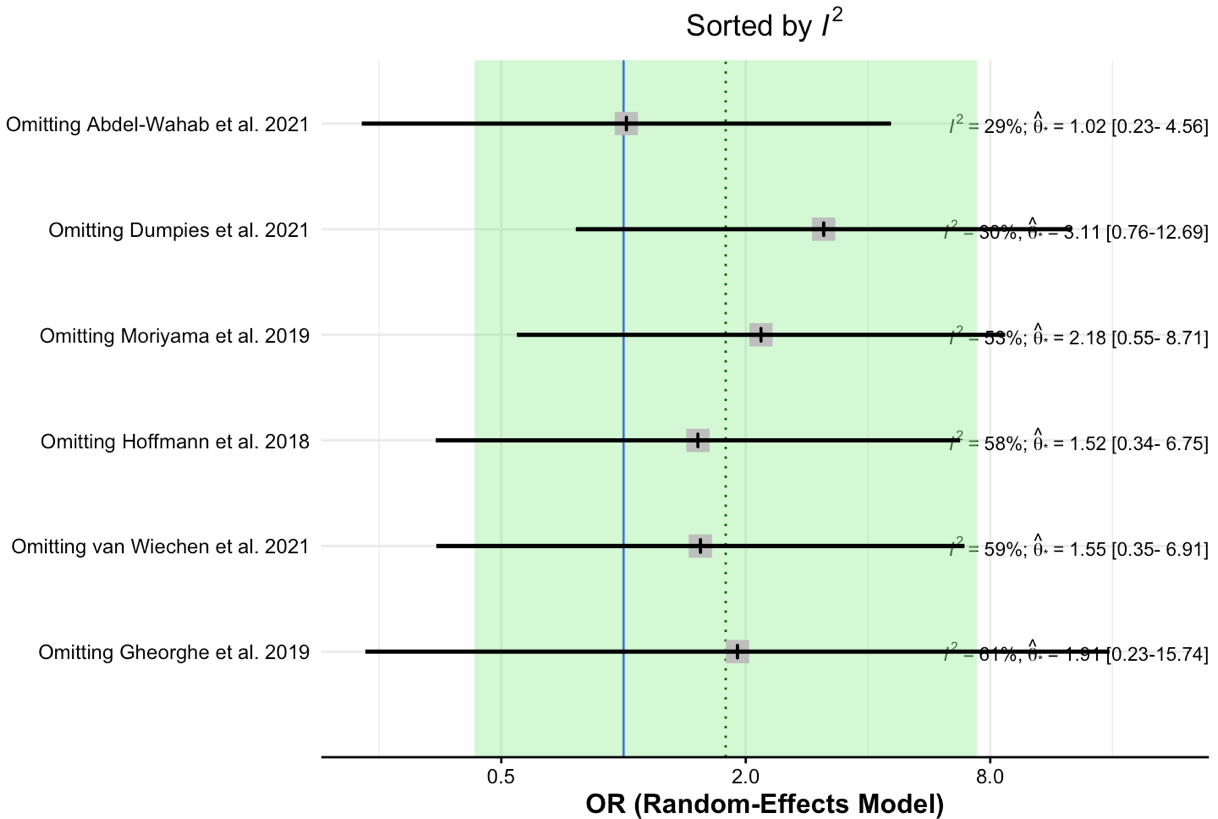
**

**Supplementary Figure 3-M: Leave-one out sensitivity analyses of requiring blood transfusion**

**
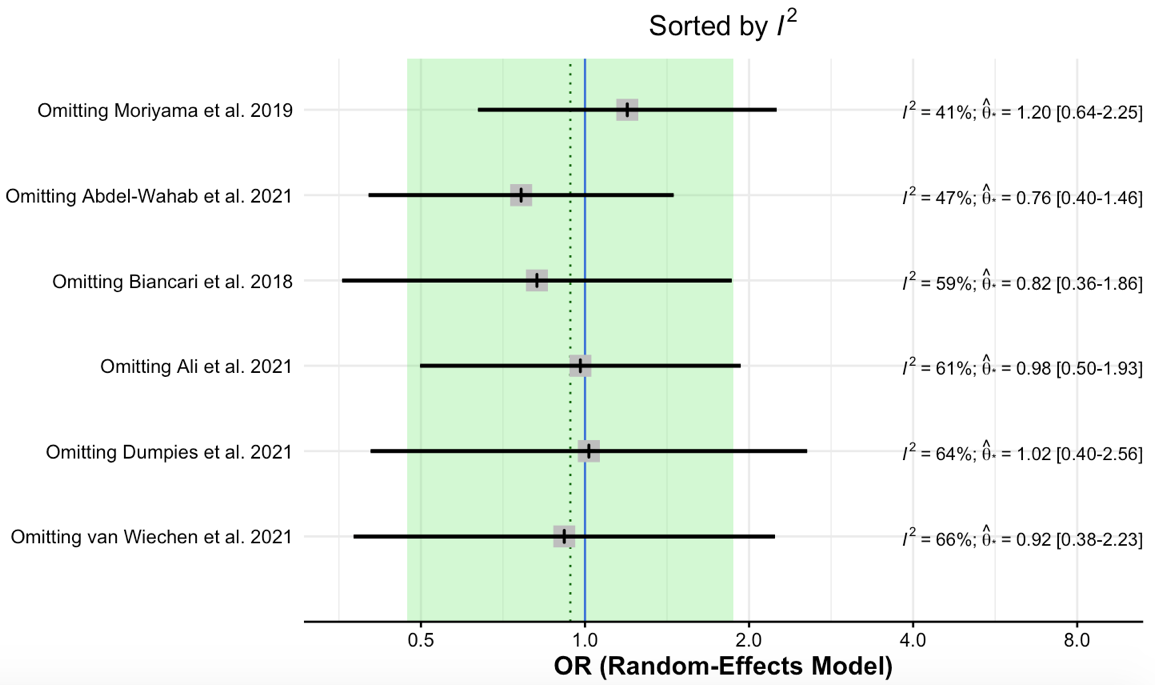
**

**Supplementary Figure 4-A: forest plot of length of stay after the procedure (MANTA^®^ versus Proglide^®^)**

**
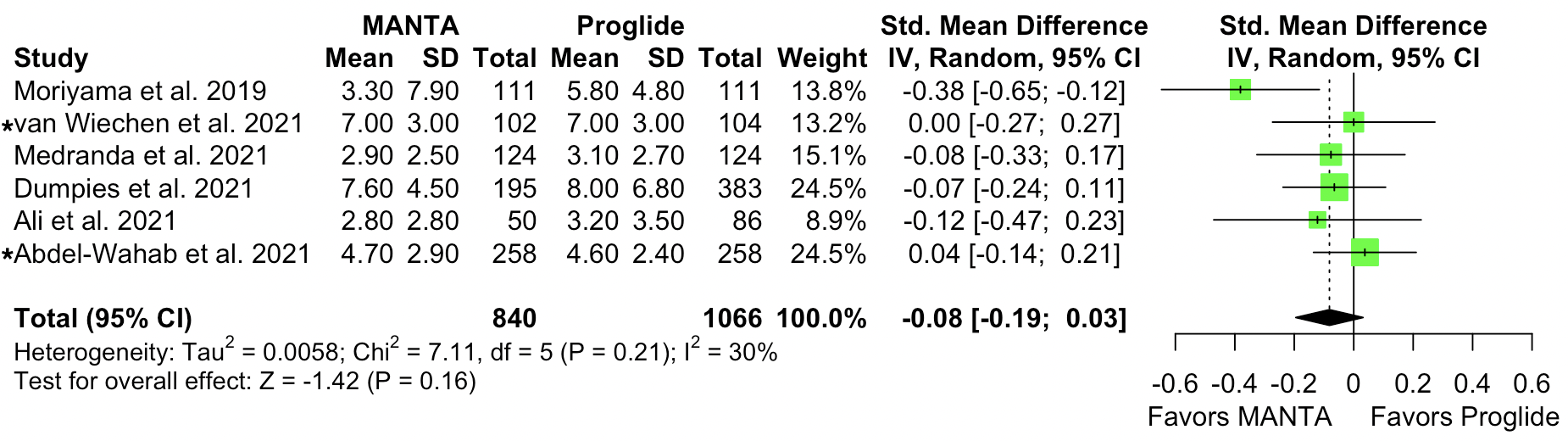
**

^* Randomized controlled trial^

**Supplementary Figure 4-B: forest plot of vascular closure device failure (MANTA^®^ versus Proglide^®^)**

**
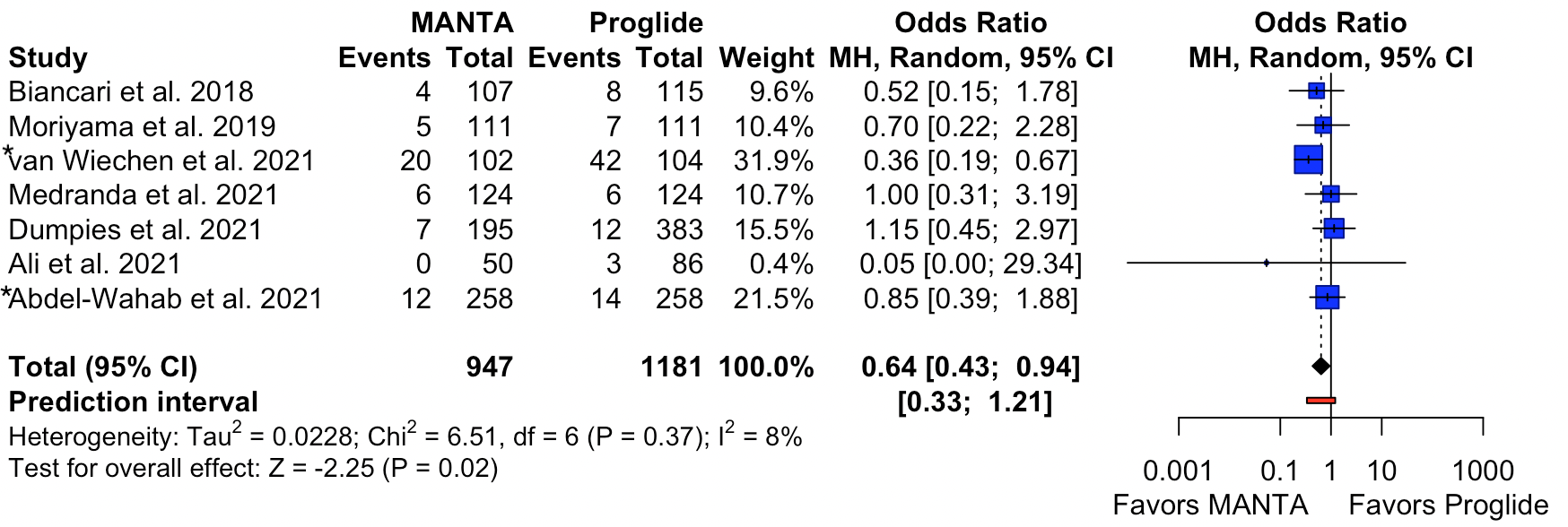
**

^* Randomized controlled trial^

**Supplementary Figure 4-C: forest plot of all-cause mortality (MANTA^®^ versus Proglide^®^)**

**
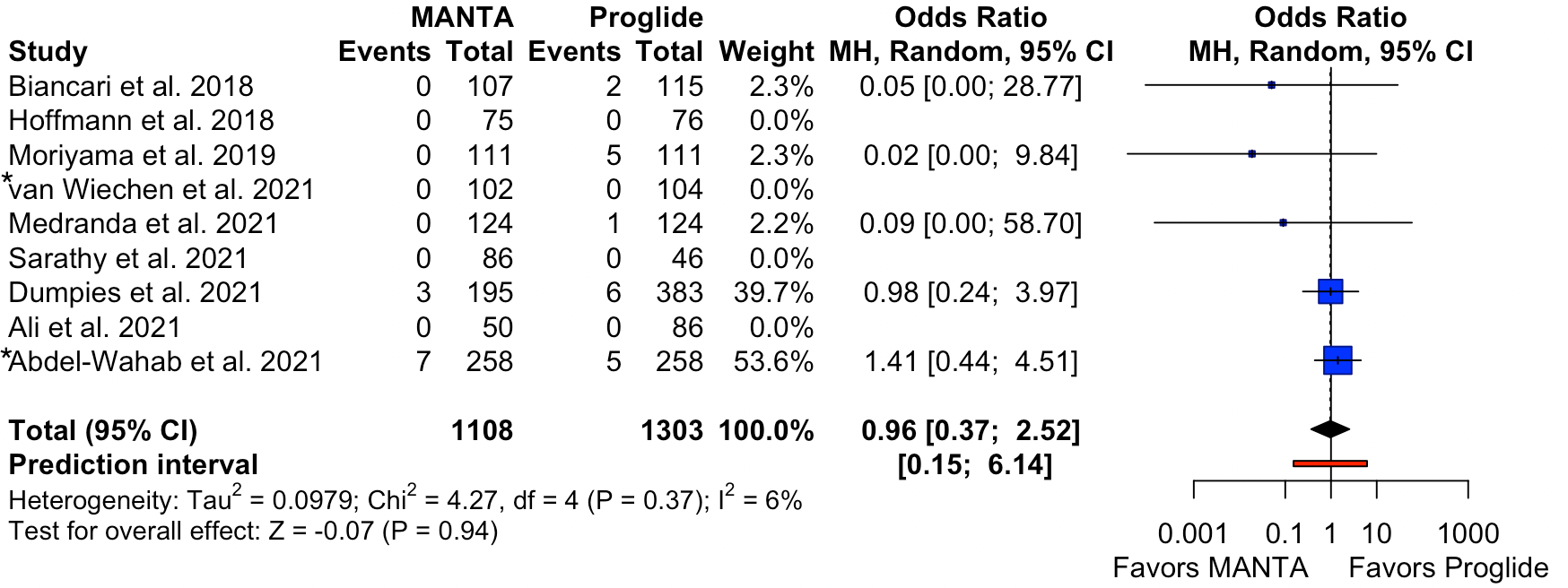
**

^* Randomized controlled trial^

**Supplementary Figure 4-D: forest plot of major bleeding (MANTA^®^ versus Proglide^®^)**

**
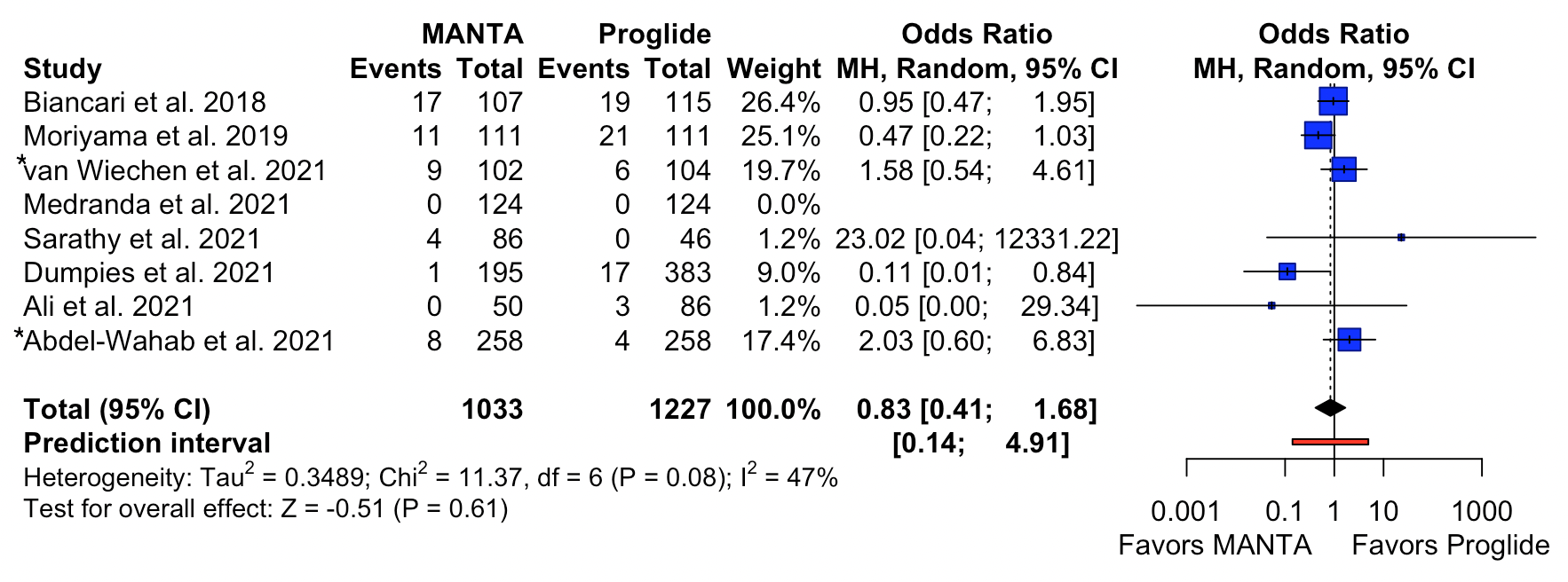
**

^* Randomized controlled trial^

**Supplementary Figure 4-E: forest plot of minor bleeding (MANTA^®^ versus Proglide^®^)**

**
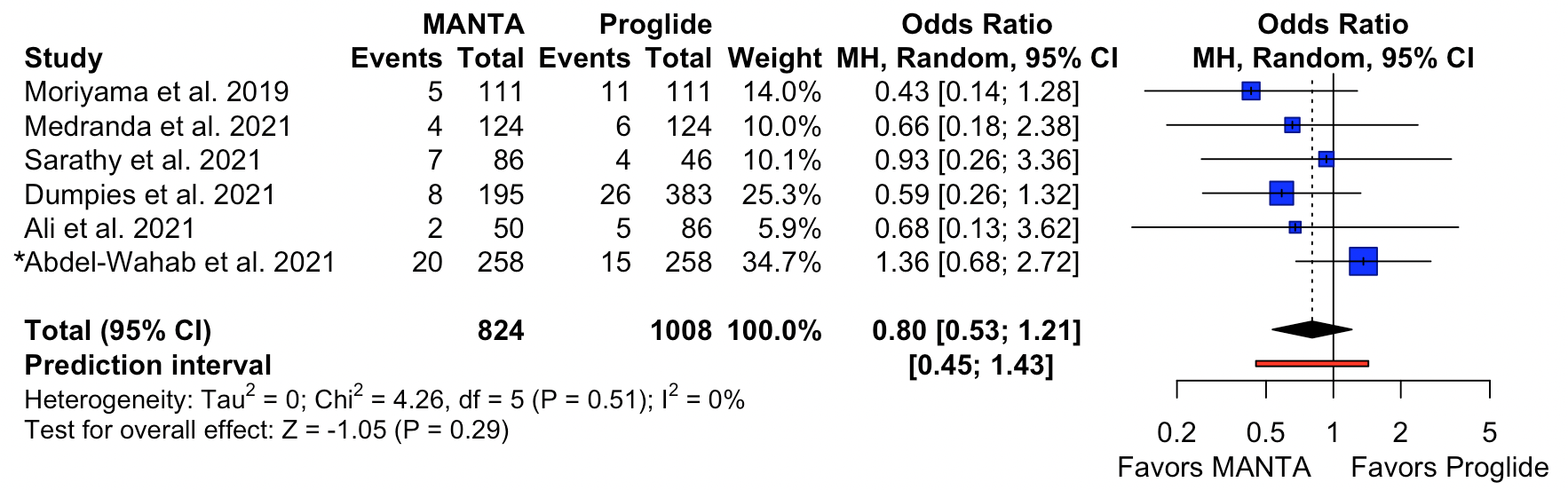
**

^* Randomized controlled trial^

**Supplementary Figure 4-F: forest plot of major vascular complications (MANTA^®^ versus Proglide^®^)**

**
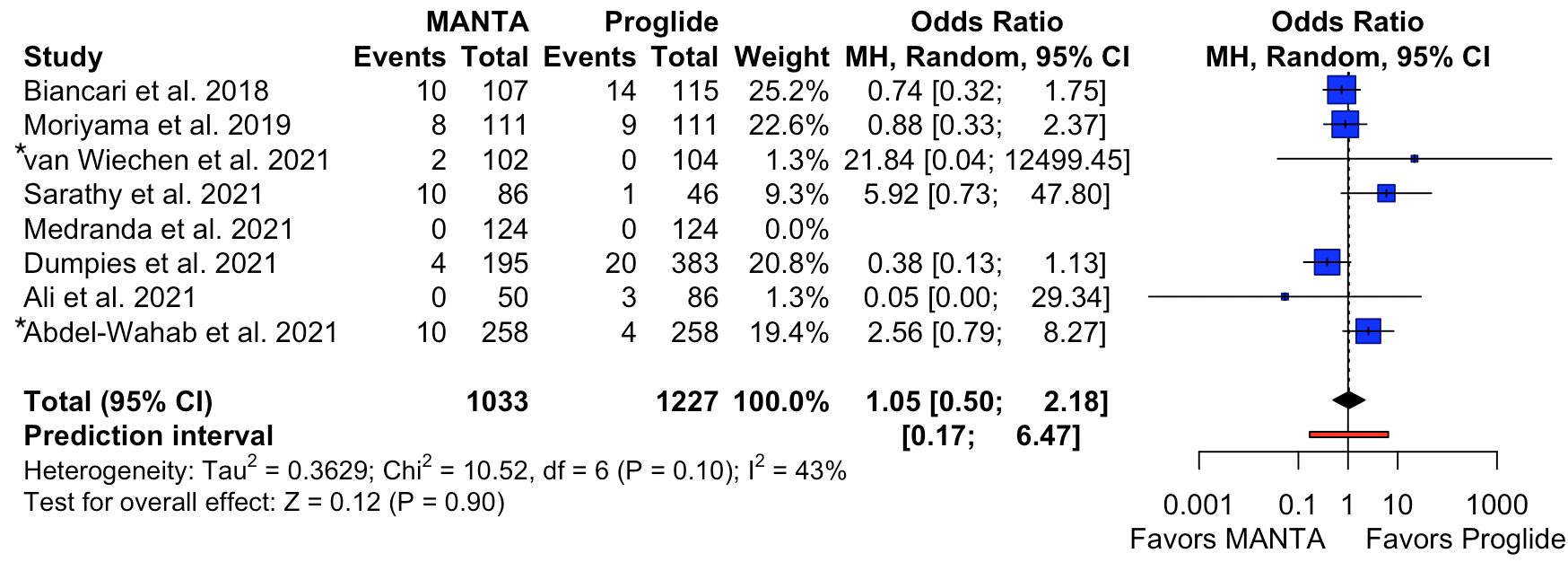
**

^* Randomized controlled trial^

**Supplementary Figure 4-G: forest plot of minor vascular complications (MANTA^®^ versus Proglide^®^)**

**
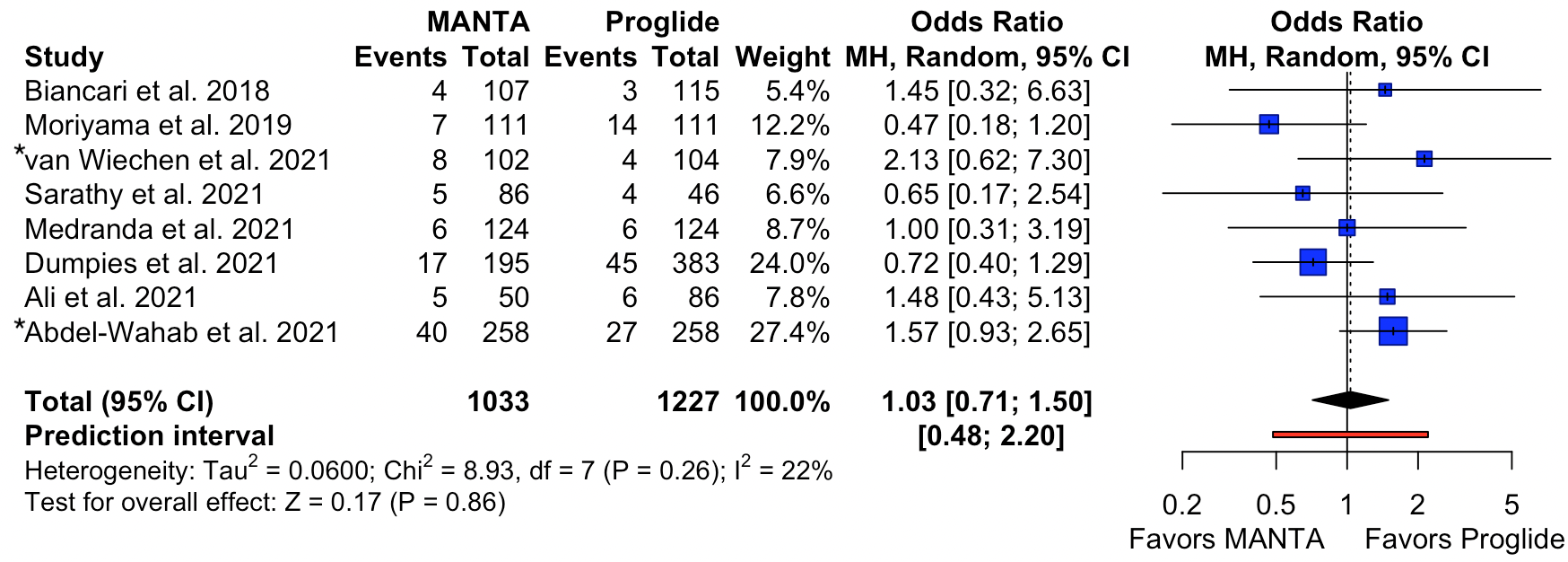
**

^* Randomized controlled trial^

**Supplementary Figure 4-H: forest plot of hematoma (MANTA^®^ versus Proglide^®^)**


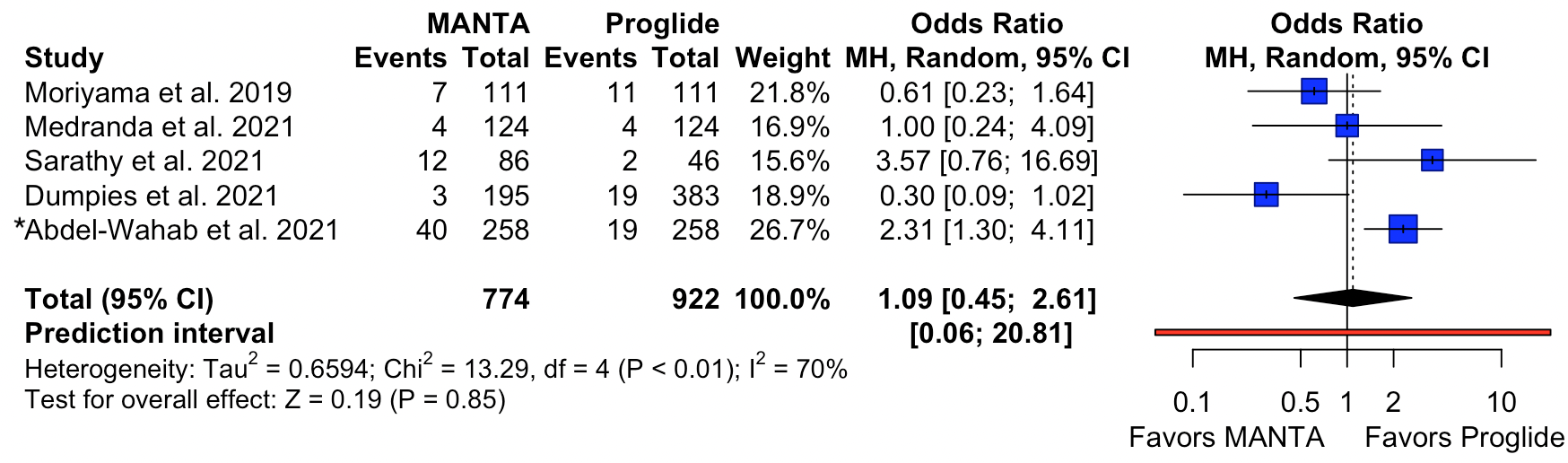


^* Randomized controlled trial^

**Supplementary Figure 4-I: forest plot of flow limiting dissection (MANTA^®^ versus Proglide^®^)**

**
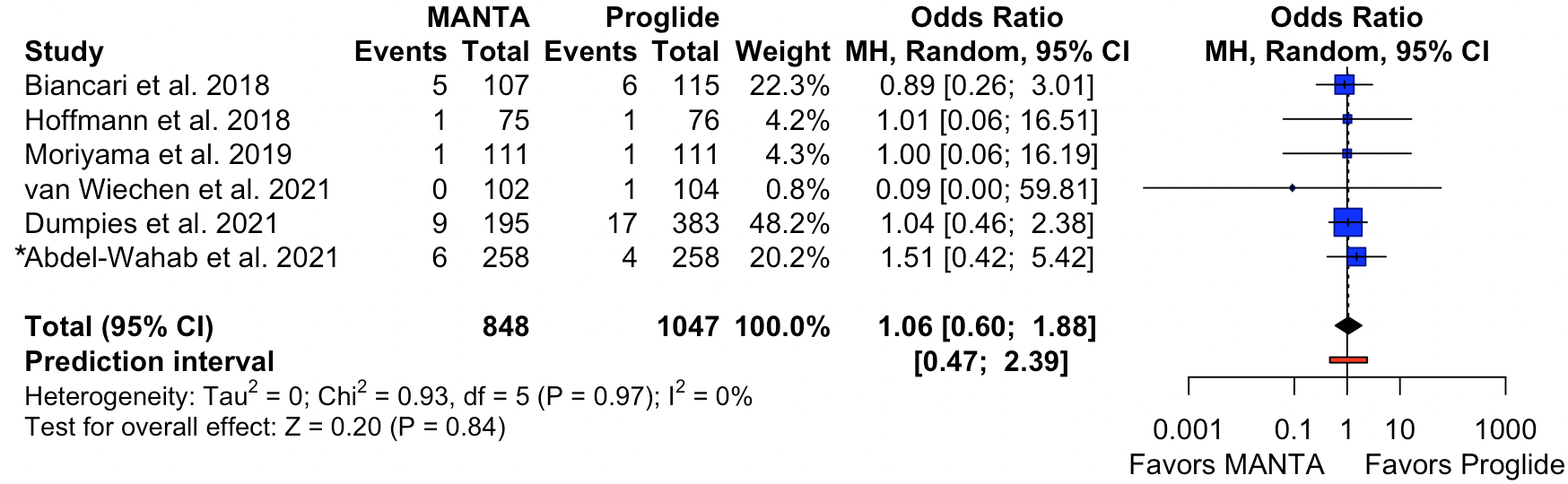
**

^* Randomized controlled trial^

**Supplementary Figure 4-J: forest plot of flow limiting vascular occlusion (MANTA^®^ versus Proglide^®^)**


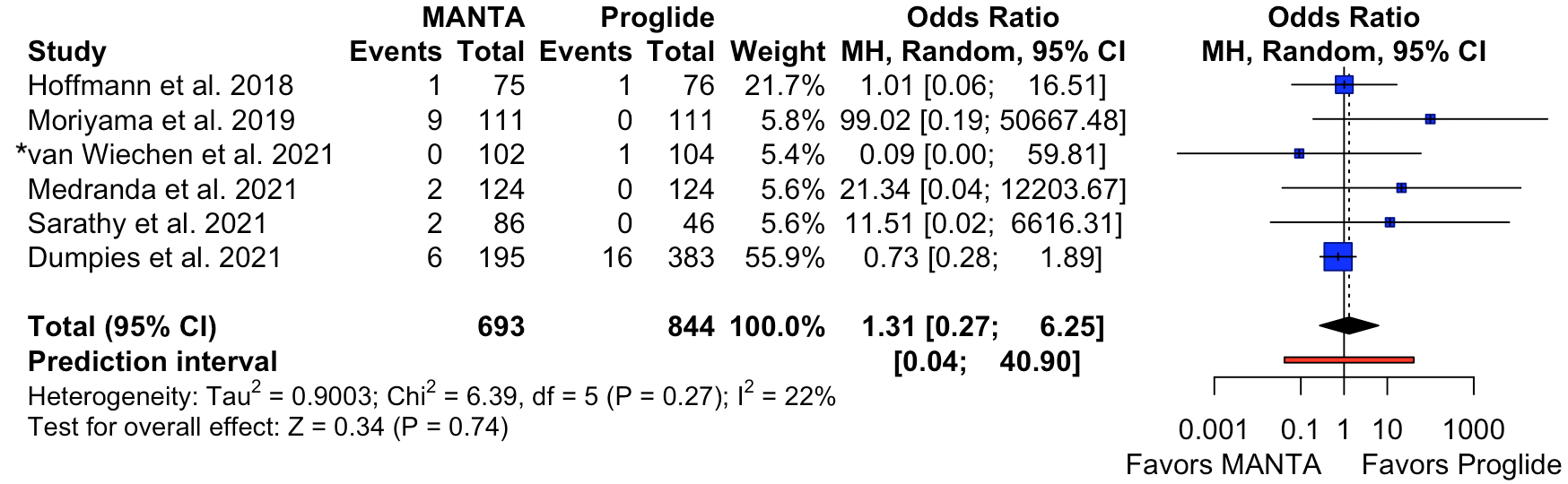


^* Randomized controlled trial^

**Supplementary Figure 4-K: forest plot of pseudoaneurysm (MANTA^®^ versus Proglide^®^)**

**
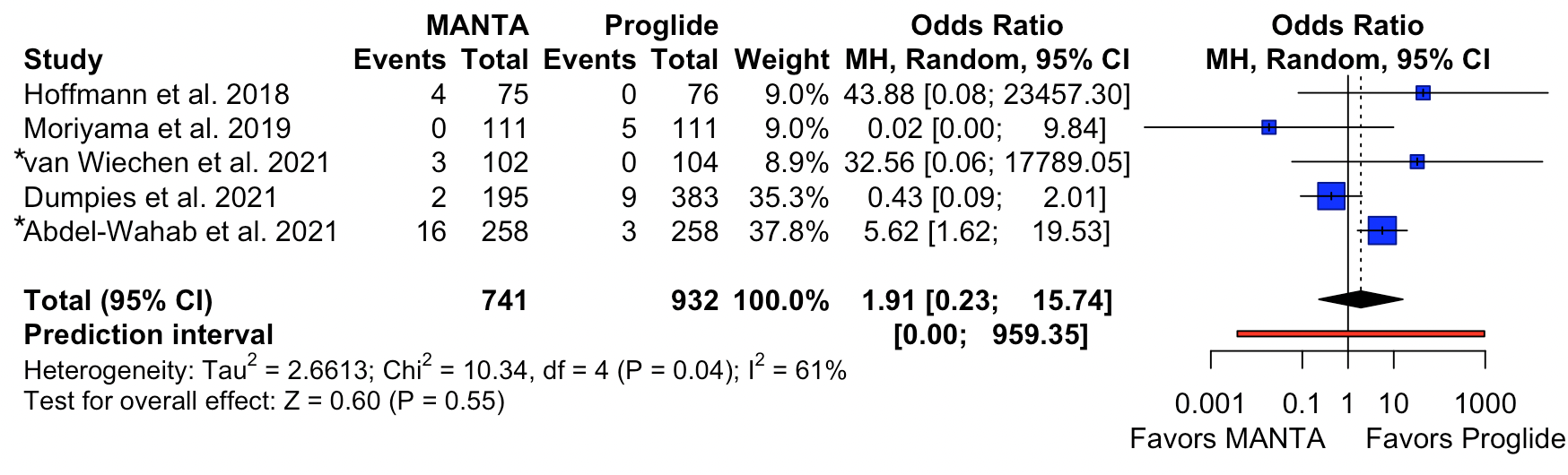
**

^* Randomized controlled trial^

**Supplementary Figure 4-L: forest plot of requiring bleed transfusion (MANTA^®^ versus Proglide^®^)**


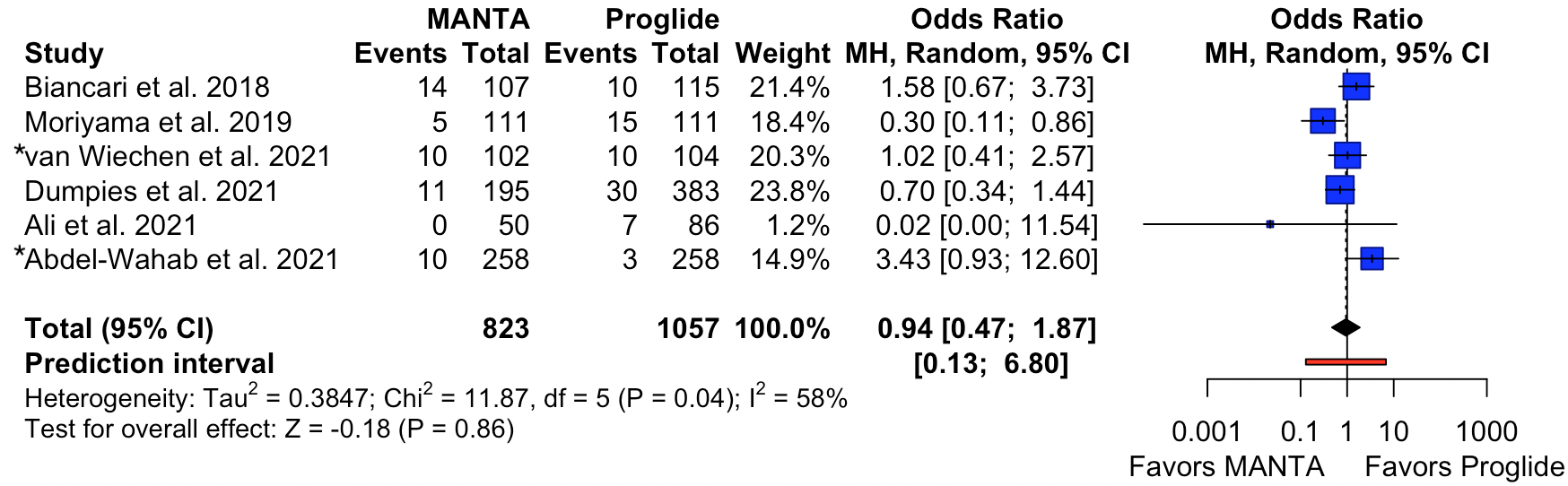


^* Randomized controlled trial^

**Supplementary Figure 4-M: forest plot of additional vascular interventions (MANTA^®^ versus Proglide^®^)**

**
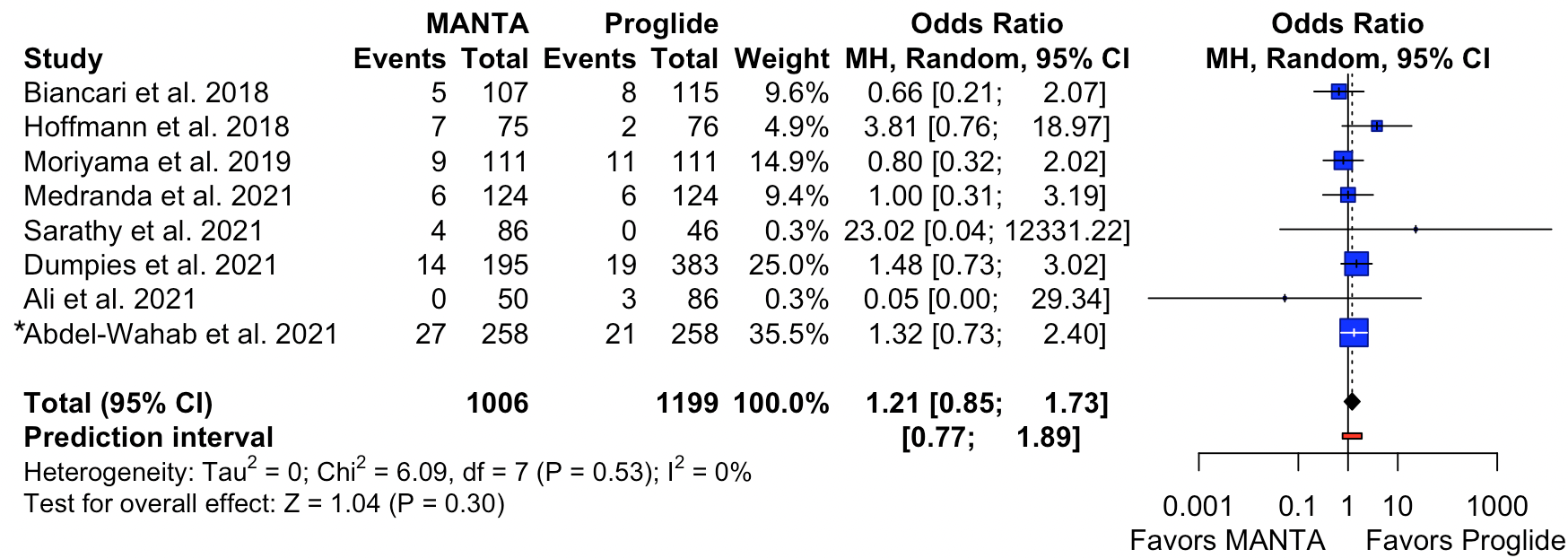
**

^* Randomized controlled trial^
